# An exposure to endocrine active persistent pollutants and endometriosis — a review of current epidemiological studies

**DOI:** 10.1007/s11356-022-24785-w

**Published:** 2022-12-23

**Authors:** Dorota Szczęsna, Katarzyna Wieczorek, Joanna Jurewicz

**Affiliations:** 1 Department of Chemical Safety, Nofer Institute of Occupational Medicine, St. Teresa Street 8, 91-348 Lodz, Poland; 2 Department of Toxicology, Medical University of Lodz, Muszyńskiego 1A Street, 90-151 Lodz, Poland

**Keywords:** Endocrine-disrupting chemicals, Endometriosis, Environmental exposure, Risk factor

## Abstract

Widespread exposure to persistent pollutants can disrupt the bodies’ natural endocrine functions and contribute to reproductive diseases like endometriosis. In this review, we focus at the relationship between endocrine-disrupting chemicals (EDCs), including metals and trace elements, organochlorine pesticides (OCPs), polychlorinated biphenyls (PCBs), polybrominated biphenyls (PBBs), poly-brominated diphenyl ethers (PBDEs), polychlorinated dibenzodioxin (PCDDs), polychlorinated dibenzofurans (PCDFs), and per- and polyfluoroalkyl substances (PFAS) exposure and risk of endometriosis. Relevant studies from the last 10 years by November 2022 were identified by searching Pubmed, Web of Science, and Scopus. The cohort and case–control studies that reported effect size with 95% confidence intervals (CIs) of EDC exposure and endometriosis were selected. Twenty three articles examining the relationship between endometriosis and exposure to persistent EDCs were considered. Most of the studies indicated association with exposure to persistent chemicals and development of endometriosis. The consistent results were found in case of lead, PCB-28, PCB-138, PCB-153, PCB-180, PCB-201, 1,2,3,7,8 — PeCDD, 2,3,4,7,8 — PeCDF and all described OCPs, showing the increased risk of endometriosis. These results support that exposure to certain EDCs, including OCPs, PCBs, PBBs, PBDEs, PFAS, and lead increase the risk of endometriosis.

**Supplementary Information:**

The online version contains supplementary material available at 10.1007/s11356-022-24785-w.

## Introduction

Every day, people are exposed to chemicals and contaminants. Pollutants generated from rapidly developing agricultural and industrial sectors are ubiquitous. The major worldwide problem generates the persistent pollutants because they are resistant in the environment conditions, can fast and easily migrate for a long distance with air and water, and readily bioaccumulate in human tissues (Ren et al. 2018). Persistent organic pollutants (POPs) are characterized by long half-lives in soils, air, sediments, or biota (years or decades). They possess strong hydrophobic and lipophilic properties (Lind and Lind 2020). The group of POPs includes such compounds like: chemicals with industrial and technical use (polychlorinated biphenyls (PCBs), polybrominated diphenyl ethers (PBDEs), and perfluorooctanesulfonate (PFOS)); pesticides (organochlorine pesticides (OCPs) such as dichlorodiphenyltrichloroethane (DDT) and its metabolites); and residual material from the industrial processes (polychlorinated dibenzo-p-dioxins (PCDDs), polychlorinated dibenzofurans (PCDFs), and polyaromatic hydrocarbons (PAHs)) (Nisha et al. 2018). PAHs do not strictly belong to POPs, but they are frequently classified as POPs in many studies, since they are metabolized by most living systems (UNECE 1998; Alharbi et al. 2018). The POPs are able to easily bioaccumulate especially in human fatty tissue. Their persistence in the environment and in the human body make POPs a major threat to human health (Lind and Lind 2020). Epidemiological studies have shown also a negative impact of exposure to metals and trace elements on reproductive health and endocrine system that metals and trace elements are also suspected of being endocrine disruptors (Diamanti-Kandarakis et al. 2009). The exposure to persistent chemicals is associated with adverse health effects such as endocrine disruption, reproductive problems, cancer, cardiovascular disease, obesity, and diabetes (Cabrera-Rodriguez 2019; Papadopoulou 2013). Endocrine disrupting chemicals (EDCs) are substances in the environment (air, soil, or water supply), food sources, personal care products, and manufactured products that interfere with the normal function of your body’s endocrine system (Endocrine Society 2022). A report by the United Nations Environmental Program (UNEP) and World Health Organization (WHO) highlighted 800 chemicals as potential EDCs (UNEP 2013). Endocrine disruptor chemicals are intensively examined due to their estrogenic, androgenic, anti-androgenic, and antithyroid activity (Arnold et al. 1997; Guillette 2006; Kristensen et al. 2011). Several classes of EDCs act as anti-androgens (p-DDT) and as thyroid hormone receptor agonists or antagonists (PCBs), and more recently, androgenic and estrogenic EDCs have been identified (PBDEs, PCBs, DDT) (Kelce and Gray 1998; Zoeller 2021; Amir et al. 2021). Heavy metals also indicate hormonal activity, suggesting that these compounds are EDCs (Pérez-Debén et al. 2020; Baltaci et al. 2019; Mitra et al. 2017). The EDCs may cause endocrine disruption in the hypothalamic-pituitary–gonadal pathway and cerebral-pituitary-thyroid pathway or affect nerve transmitters (neurotransmitters) in the central nervous system. Crucial adverse effect on the hormonal system has an acrylic hydrocarbon receptor (AhR) which acts in regulation of the cytochrome P-450 induction. EDCs can bind this receptor consequently and many side additional biological reactions are triggered at the level of the whole body (Safe et al. 1998; Guo et al. 2019). One of the effects of exposure to these chemicals is the adverse impact on the reproductive system including poor fertility and pregnancy outcome (Vested et al. 2014). According to the WHO (World Health Organization) report, 10% (190 million) of reproductive age women and girls suffer from endometriosis (Zondervan 2020). The current literature confirms the existence of a link between persistent EDC exposure and the development of endometriosis (Rumph et al. 2020). The first report about the connection between EDCs and endometriosis was noticed after the application of diethylstilbestrol (DES). Daughters of women taking DES to prevent miscarriages and preterm birth in pregnant (from 1938 to 1971) had increasing odds for progressing endometriosis and infertility later in life (Schechter et al. 2005; Stillman and Miller 1984). DES has not harmful effect on pregnancy outcomes.

Endometriosis is a disease characterized by the presence of tissue resembling endometrium (the lining of the uterus) outside the uterus (WHO 2018). Endometriosis has histological subtypes like ovarian endometrioma (OvE), deep infiltrating endometriosis (DIE), and peritoneal endometriosis (Bordonné et al. 2021). Endometriosis occurs in 5–10% of women of childbearing age, 50–60% of women and teenagers with pelvic pain and up to 50% of women with infertility (Zondervan et al. 2018). Endometriosis is more common in Caucasians than African Americans and Asians, in taller women with lower BMI and genetic factors increase its risk by 50% (Chatman 1976). It is the most common cause of deep dyspareunia, pelvic pain, and reduced fertility (Ota et al. 2018). Numerous articles indicate association with exposure to persistent EDCs and development of endometriosis. This review is a summary of the last 10 years studies at the connection between persistent environmental pollutants with endocrine disruption properties and endometriosis risk.

## Materials and methods

### Literature search methodology — strategy and eligibility criteria

The PRISMA (Preferred Reporting Items for Systematic reviews and Meta-analyses extension for Scoping Reviews (PRISMA-ScR) was employed to guide this review (Tricco et al. 2018). A literature search of relevant papers was conducted from the beginning of 2012 to November 2022 (10 years) using the electronic bibliographic database Pubmed, Web of Science, and Scopus. Only original articles were included. The period was chosen to reflect findings over the past 10 years, during which the availability of sensitive, specific, and affordable bioassays made biomarkers feasible for use in epidemiological studies for measuring exposures to those compounds. Additionally, the period 2012–2022 was chosen because there were only few studies conducted on endometriosis risk and exposure to persistent environmental EDCs prior to 2012. The search strategy included the keywords in the title or abstract. The search combined terms referring to the exposure to environmental EDCs and endometriosis. The combinations of the keywords used were as follows:

1. Those referring to the exposure: environmental exposure to persistent endocrine disrupting chemicals, exposure to organochlorine pesticides (OCPs), polybrominated diphenyl ether (PBDEs), polychlorinated biphenyls (PCBs), polybrominated biphenyls (PBBs), polychlorinated dibenzo-p-dioxins, and to polychlorinated dibenzofuransphthalates, and metals (cadmium, lead, mercury, copper, nickel, zinc and chromium).
2. Those referring to the outcome: endometriosis.

Relevant studies were also identified through a review of the references cited in all the published studies. In total, 340 articles were found as the result of the search, and they were checked for eligibility. The review included peer-review studies on the effect of exposure to environmental persistent endocrine disrupting chemicals and endometriosis risk. Studies that analyzed the association of environmental exposure to non-persistent pollutants as well as those assessing the occupational exposure and animal and in vitro studies were excluded. The exclusion criteria were also a study published before 2012 and publication in a different language than English. The cohort and case–control studies that reported effect size with 95% confidence intervals (CIs) of EDC exposure and endometriosis were selected. Finally, 23 publications on the effect of exposure to persistent endocrine disrupting chemicals and endometriosis were selected by two reviewers with agreement (*k* = 0.82).

### Study selection

All the related data was extracted independently by 2 reviewers independently assessing which ones should be included in this review, and incongruences were resolved by discussion and the intervention of a third independent author. Irrelevant studies were excluded. The remaining articles were subject to a full-text review. All the full-text articles were thoroughly examined to identify the aims of the studies, type of epidemiological study, statistical methods, and accurate results. Finally, for the purpose of this review, from each study the following information was abstracted: study population; type of epidemiological study; and type of exposure and methods used for its analysis and assessment (including biomarkers).

## Results

### Metals and trace elements

Metals are chemically stable, naturally occurring elements and tend to persist in human tissues as well as in the environment. Because of their low trace concentrations in the range of ppb (but less than 10 ppm), they are also considered as trace elements (Kabata-Pendias 2000). Metals are used in agriculture, domestic, industry, and technological applications. The general population is exposed mainly through consumption of contaminated drinking water and food (Fu and Xi 2020). Metals, such as lead, mercury, and cadmium accumulation in the human body affects metabolism and cause a wide spectrum of reproductive and developmental adverse effects by induction of reactive oxygen species (ROS) which contributes to the oxidative stress (Rehman et al. 2018; Jaishankar et al. 2014). Metals can act as carcinogens and mutagens, cause neurological disorders, damage kidney function, and cause other endocrine abnormalities (Nieder et al. 2018).

Recently, plenty of research has been focused on the endocrine toxicity of selected metals, trace elements, and their relationship with endometriosis. Metals and trace elements are suspected of being endocrine disruptors (UNEP 2013). Epidemiological studies have shown a negative impact of exposure to those chemicals on reproductive health and endocrine system including occurrence of endometriosis (Dutta et al. 2021). Role of heavy metals and trace elements in development of endometriosis has not been explained yet. One of the proposed hypotheses is that the phagocytic cells, main producers of both ROS, and reactive nitrogen species (RNS) are enrolled and activated by pro-inflammatory and chemotactic cytokines and probably related in endometriosis progress (Mier-Cabrera et al. 2010).

Seven epidemiological studies have indicated associations between the exposure to selected metals and endometriosis (Zhang et al. 2021; Lai et al. 2017; Tanrikut et al. 2014; Pollack et al. 2013; Kim et al. 2021; Silva et al. 2013, Yılmaz et al. 2020). Studies mostly assess cadmium and lead exposure (six of them) (Zhang et al. 2021; Lai et al. 2017; Tanrikut et al. 2014; Pollack et al. 2013; Kim et al. 2021; Silva et al. 2013), copper (three of them) (Lai et al. 2017, Pollack et al. 2013, Yılmaz et al. 2020), and mercury (four of them) (Zhang et al. 2021; Lai et al. 2017; Tanrikut et al. 2014; Pollack et al. 2013) exposure. Other metals such as nickel (Silva et al. 2013; Yılmaz et al. 2020), zinc (Lai et al. 2017; Yılmaz et al. 2020), manganese, iron (Lai et al. 2017), aluminum (Yılmaz et al. 2020), and chromium (Lai et al. 2017; Pollack et al. 2013) also have been studied in relation to endometriosis. Most of the studies were performed in Asia (Taiwan, Sri Lanka, Turkey, Korea, China) (Zhang et al. 2021; Lai et al. 2017; Tanrikut et al. 2014; Kim et al. 2021; Silva et al. 2013) and the remaining in the USA (Pollack et al. 2013; Yılmaz et al. 2020). Four studies have been classified as case–control (Tanrikut et al. 2014; Pollack et al. 2013; Silva et al. 2013; Yılmaz et al. 2020), two as cross-sectional (Zhang et al. 2021; Lai et al. 2017), and one as cohort (Kim et al. 2021). Women in most of studies were recruited in fertility and gynecology centers (Lai et al. 2017; Pollack et al. 2013; Silva et al. 2013; Yılmaz et al. 2020), in one study female workers exposed to lead was recruited from Korean Occupational Safety and Health Agency (Kim et al. 2021), one study was based on the National Health and Examination Survey (NHANES) (Zhang et al. 2021), and one was conducted at university (Tanrikut et al. 2014). Two authors investigated the associations between endometriosis, exposure to metals, and infertility (Lai et al. 2017; Tanrikut et al. 2014). Tanrikut et al. recruited 65 volunteers with endometriosis including 33 infertility women and 32 fertile women. Lai et al. analyzed 190 samples from infertile women, where 68 cases had endometriosis. The age of women participating in the studies ranged from 18 to 70 years. Endometriosis was confirmed by surgical visualization (Tanrikut et al. 2014; Pollack et al. 2013), pelvic magnetic resonance imaging (MRI) (Pollack et al. 2013), the FIGO classification (International Federation of Gynecology and Obstetrics — positive histology confirmed endometriosis) (Yılmaz et al. 2020), laparoscopic surgery (Lai et al. 2017; Silva et al. 2013), principle diagnosis (first time hospital admission for endometriosis) (Kim et al. 2021), and questionnaire (Zhang et al. 2021). In presented studies, metals and trace elements have been widely detected in various biological fluids including urine (Pollack et al. 2013), serum (Yılmaz et al. 2020), blood (Zhang et al. 2021; Lai et al. 2017; Kim et al. 2021; Silva et al. 2013), and endometrial tissues (Tanrikut et al. 2014).

#### Exposure to mercury

The association between mercury and endometriosis was investigated in four studies (Zhang et al. 2021; Lai et al. 2017; Tanrikut et al. 2014; Pollack et al. 2013). The details of these studies are provided in the Supplementary Materials (Table S1). Only one study in recent 10 years conducted on the US population indicates a threefold higher association between Hg and endometriosis in the second tertile versus the first tertile (*OR*: 2.77; 95% *CI*: 1.47–5.47) (Zhang et al. 2021). Two studies performed among 190 infertile Taiwanese women (Lai et al. 2017) and 33 infertile Turkish women (Tanrikut et al. 2014) observed no relationship between presence of mercury in blood and endometrial tissue with occurrence of endometriosis. Additionally, in the study performed by Tanrikut et al., the measurement of endometrial tissue Hg concentration indicates lack of this heavy metal in biological samples from either group with endometriosis (33 cases with unexplained infertility and 32 controls with no history of infertility). In the study performed by Pollack et al., no association was found between exposure to mercury and endometriosis among 473 women (190 with endometriosis, without endometriosis 283) with surgically visualized disease (operative cohort) and among women 131 women including 14 with endometriosis, 113 without endometriosis but with magnetic resonance confirmation of diagnosis (population cohort) (Pollack et al. 2013).

#### Exposure to cadmium

The association between cadmium and endometriosis was investigated in six studies (Zhang et al. 2021; Lai et al. 2017; Tanrikut et al. 2014; Pollack et al. 2013; Kim et al. 2021; Silva et al. 2013). The details of these studies are provided in the Supplementary Materials (Table S2). The highest significant increases in the odds of an endometriosis diagnosis have been reported for cadmium in 65 Turkish women with surgically visualized disease including 33 women with unexplained infertility and 32 fertility women. (*OR*_Adjusted_: 18.9; 95% *CI*: 4.5, 79.9). Blood cadmium level associated with significant reduction in the odds in the study performed among women from operative (endometriosis was confirmed surgically, *N* = 473 women) and population cohort (endometriosis was confirmed by MRI, *N* = 131) was described by Pollack et al. (2013) (*OR*_Adjusted_: 0.28; 95% *CI*: 0.25, 0.31; *OR*_Adjusted_: 0.34; 95% *CI*: 0.31, 0.37 — for geometric mean distributions of trace elements by endometriosis status irrespective of cohort). Moreover, the same authors investigated the correlations between concentration of metals Cd, Cr, and Cu in urine decreasing odds ratio for cadmium (as well as for chromium which is described below) *OR*_Adjusted_ = 0.52; 95% *CI*: 0.29, 0.93 — lowest tertile versus women in the highest tertile and third versus first tertile: *OR*_Adjusted_ = 0.55; 95% *CI*: 0.31, 0.98 — diagnosis irrespective of statistical model. Jackson et al. (2011) reported that cigarette smoking (predictor of cadmium exposure) is associated with a lower odds of endometriosis (*OR*_Adjusted_ = 0.50; 95% *CI*: 0.3, 0.9) (Jackson et al. 2011).

In contrast to these findings, the authors of four studies (Zhang et al. 2021; Lai et al. 2017; Kim et al. 2021; Silva et al. 2013) did not find an association between exposure to cadmium and endometriosis. No relationship was reported by Zhang et al. (2021) among 77 American women, where endometriosis status was self-reported via questionnaire (1127 control group) (Zhang et al. 2021). Kim et al. (2021) observed no association between exposure to cadmium and endometriosis confirmed by principal diagnosis among 26,542 women (Kim et al. 2021). In the study conducted by Lai et al. (2017) including 190 infertile women with laparoscopic surgery confirmation of endometriosis, also no relationship was found (Lai et al. 2017). Moreover, no association was observed in a study published by Silva et al. (2013) in a group of hundred women with visually diagnosed endometriosis, where cases had lower blood cadmium levels, as compared to controls, and results were not statistically significant (Silva et al. 2013). Cadmium toxicity focusing on altered cell adhesion and signaling, increased oxidative damage and apoptosis, DNA damage, ionic and molecular mimicry, cell cycle disturbance, and epigenetic alterations. Some authors reported that women with unexplained infertility have high endometrial cadmium concentrations. It can suggest that endometrial dysfunction and implantation failure may be caused by cadmium (Rzymski 2015).

#### Exposure to lead

Exposure to lead and endometriosis was assessed in six studies (Zhang et al. 2021; Lai et al. 2017; Tanrikut et al. 2014; Pollack et al. 2013; Kim et al. 2021; Silva et al. 2013). The details of these studies are provided in the Supplementary Materials (Table S3). Two studies examine the association between blood lead level and endometriosis, and both reported the increase in the odds of a disease diagnosis (Kim et al. 2021; Silva et al. 2013). One of the studies investigated the relationship between lead exposure and blood lead levels (BLLs) and endometriosis admission (Kim et al. 2021). To compare endometriosis admission caused by lead exposure and blood lead levels (BLLs) in the lead-exposed group (study group), multiple comparisons were performed using an external control group (general population) and internal control group (noise-exposed group). Only in the internal control group — noise exposed group (*OR*_Adjusted_ = 1.48; 95% *CI*: 1.11, 1.98 for group women with BLL < 5 g/dL), the increasing odds was observed. Also, the association between endometriosis and co-exposure to lead and cadmium was observed among cohort of lead-exposed female workers (*OR*_Adjusted_: 1.39; 95% *CI*: 1.14–1.67). Another cross-sectional study reported statistically significant odds ratio between EM and exposure to Pb (*OR*_Adjusted_: 2.59; 95% *CI*: 1.11, 6.06 — the third tertile versus first tertile) (Lai et al. 2017). The study group included 68 patients with endometriosis (cases) and 122 women without endometriosis (controls); all women were infertile. Endometriosis diagnosis was confirmed by laparoscopic surgery and also proved by pathology. Whereas, in four studies no association was found between exposure to lead and endometriosis. In one case–control study performed in Sri Lanka, among 50 women with endometriosis diagnosed visually — subsequent to laparotomy or laparoscopy (50 controls) the higher blood lead level was observed among cases compared to controls but *P* value was not statistically significant (Silva et al. 2013). In another case–control study among 33 infertility Turkish women and 32 fertile women, lead was detected only in five from 33 participants (15% detection frequency for case group) and in only one from 32 women (control group) (Tanrikut et al. 2014). Likewise, analysis conducted among 77 women with endometriosis recruited from NHANES study (National Health and Nutrition Examination Survey) in US population (2001–2006) did not show significant association between EM and exposure to Pb (Zhang et al. 2021). Pollack et al. (2013) presented that blood lead level was associated with a reduced odds of diagnosis irrespective of statistical model, but this finding was not significant when adjusting for potential confounders (*OR*_Adjusted_ = 0.84; 95% *CI*: 0.50, 1.41) (Pollack et al. 2013).

#### Exposure to copper

Three reports assess the exposure to copper and endometriosis risk (Lai et al. 2017; Pollack et al. 2013; Yılmaz et al. 2020). The details of these studies are provided in the Supplementary Materials (Table S4). The increasing odds in endometriosis risk have been found only in the study performed by Pollack et al. (2013). The authors found an increased risk of endometriosis in the third versus first tertile of exposure to copper (*OR*_Adjusted_ = 2.66; 95% *CI*: 1.26, 5.64) (Pollack et al. 2013). Another study was performed by Yılmaz et al. (2020) among a group of 40 women from which 21 used copper intrauterine device (Cu IUD) — in the past, longer than 6 months ago, and occurrence of endometrial polyps. No statistical difference neither in serum Cu levels in study and control groups or in samples from women with respect to the Cu IUD use history (Yılmaz et al. 2020). The authors also described the Cu/Zn ratio which was significantly higher in the study group when compared with the control group (*IQR*: 0.547 — control; *IQR* — study: *IQR*: 0.442). Additionally, in a cross-sectional study conducted by Lai et al. (2017) in a group of 190 infertile women no association between endometriosis and exposure to copper was found (Lai et al. 2017).

#### Exposure to chromium

Exposure to chromium and endometriosis was assessed in two studies (Lai et al. 2017; Pollack et al. 2013). The details of these studies are provided in the Supplementary Materials (Table S5). Chromium was associated with the occurrence of endometriosis in one study conducted in the US population categorized into tertiles (*OR*_Adjusted_ = 1.97; 95% *CI*: 1.21, 3.19 — second versus first tertile) (Tanrikut et al. 2014). Moreover, correlations between concentration of metals: cadmium, chromium, and copper analyzed in urine samples indicated an increasing odds ratio for chromium (second versus lowest tertile — *OR*_Adjusted_ = 2.32; 95% *CI*: 1.42, 3.79). Chromium together with copper might catalyze redox homeostasis by co-synthesis with proteins or in the production of cellular toxins so the association between these metals and endometriosis is explicit. In the second study conducted on a group of 190 infertile women by Lai et al., it found no association between chromium and endometriosis (Lai et al. 2017).

#### Exposure to zinc

Two studies assess the relationship between exposure to zinc and endometriosis (ai 2017; Yılmaz et al. 2020). The details of these studies are provided in the Supplementary Materials (Table S6). In the study performed among groups of 80 infertile Turkish women by Yılmaz et al. (2020), interquartile ratio for ratio of Cu and Zn indicates relationship with endometrial polyps: *IQR*: 0.442 — study; *IQR*: 0.547 — control; while no statistically significant differences were observed in the same study group for serum median levels of zinc separately: *IQR*: 0.09 — study; *IQR*: 0.18 — control (Yılmaz et al. 2020). In the second study performed by Lai et al. (2017), inverse association was observed between the zinc concentration in whole blood and the presence of endometriosis in Asian infertile women (in second tertile: *OR*_Adjusted_: 0.42; 95% *CI* 0.20, 0.92 and in third tertile: OR_Adjusted_: 0.39, 95% *CI*: 0.18, 0.88) (Lai et al. 2017).

#### Exposure to nickel

The data about the exposure to nickel and endometriosis risk are lacking and inconclusive. They were described in two studies (Silva et al. 2013; Yılmaz et al. 2020), and the details are provided in the Supplementary Materials (Table S7). In the first study conducted by Silva et al. (2013), Ni concentrations in serum were statistically higher in groups with endometriosis (*N* = 50), compared to the controls (*N* = 50) (Silva et al. 2013). On the other hand in the study performed by Yılmaz et al. (2020) lower levels of nickel in whole blood in women with endometriosis was reported (Yılmaz et al. 2020). Both studies are based on the comparison of Ni in blood among cases of endometriosis and appropriate control groups.

#### Other metals

Moreover, the blood and serum levels of other elements like iron, manganese, and aluminum were determined but no relationship with endometriosis was observed (Lai et al. 2017; Yılmaz et al.  2020). The details of these studies are provided in the Supplementary Materials (Table S8). Pollack et al. in 2013 year analyzed 19 metals and trace elements (antimony, arsenic, barium, beryllium, cadmium, cesium, chromium, cobalt, copper, lead, manganese, mercury, molybdenum, nickel, tellurium, thallium, tin, tungsten, and zinc) in urine at group of 473 cases and 131 controls. Despite chromium and copper which were described above, none of the elements showed significant association with endometriosis (Pollack et al. 2013).

In conclusion, among 21 reported and analyzed metals only six (cadmium, copper, lead, mercury, zinc, and chromium) indicated association with endometriosis. In case of exposure to mercury, cadmium, copper, and chromium, only in one study the relationship was observed. Lead exposure was associated with endometriosis in most of the presented studies on this topic. Whereas in the research on nickel and zinc, only two studies were found. In both nickel studies, the link with endometriosis was observed. In zinc exposure studies, only concentrations of nickel in serum of case and control groups were assessed, indicating higher level of exposure among cases. The diverse results between studies may be caused by differences in the selection of study groups, type of metal, and nature of biological fluids.

### Organochlorine pesticides

Organochlorine pesticides (OCPs) are chlorinated hydrocarbons (at least three atoms of chlorine) used to get rid of pests like insects, fungi, and rodents (Richardson et al. 2019). Since 1970, OCPs have been withdrawn from use, but due to their persistence in the environment, semi volatility, low solubility in water, toxicity, and lipophilicity, these residues can still be found in environmental samples and food (Ferronato et al. 2018; Huang et al. 2017). Exposure to organochlorines occurs generally via respiratory tract, skin absorption, or eating contaminated fish, milk, eggs and meat (Pastor Belda et al. 2021). Epidemiological and animal studies have shown a negative impact of organochlorine pesticides on reproductive and immune system, endocrine disruption as well as dysregulation lipid metabolism (Martyniuk et al. 2020). Some polychlorinated derivatives can induce proliferation of endometrial tissue or may affect developing uterine tissue (Sandra 2016). The precise mechanism of the endometriosis development after exposure to OCPs remains unknown.

Epidemiological studies on exposure to organochlorine pesticides and endometriosis have been described in six studies (Buck Louis et al. 2012; Upson et al. 2013; Ploteau et al. 2016, 2017; Pollack et al. 2021; Matta et al. 2022). The details of these studies are provided in the Supplementary Materials (Table S9). The positive association with endometriosis have been found in all six studies for nine OCPs (β-hexachlorocyklohexane, γ-hexachlorocyclohexane, HCB, dieldrin, trans-nonachlor, oxychlordane, cis-heptachlor, dichlorodiphenyldichloroethylene (p,p-DDE) and mirex). Three of the studies were performed in the USA (Washington, California, Virginia) (Buck Louis et al. 2012; Upson et al. 2013; Pollack et al. 2021) and three in France (Ploteau et al. 2016, 2017; Matta et al. 2022). In five studies, endometriosis was surgically confirmed (Upson et al. 2013; Ploteau et al. 2016, 2017; Pollack et al. 2021; Matta et al. 2022), and in one was based on MRI assessment (Ploteau et al. 2016). Most of the studies were designed as case–control studies (Upson et al. 2013; Ploteau et al. 2016, 2017; Pollack et al. 2021), one as clinical-based analysis (Matta et al. 2022) and one as case-sectional study (Buck Louis et al. 2012). Volunteers were recruited in clinical centers (Buck Louis et al. 2012; Pollack et al. 2021); one study was based on the Women’s Risk of Endometriosis (WREN) study and the ancillary Persistent Organic Pollutants and Endometriosis Risk (POPs) study — large integrated health care system in western Washington State [60]; in one study, data were collected as part of a population-based on clinical case–control study of endometriosis performed in France (Ploteau et al. 2016, 2017; Matta et al. 2022). The age of women participating in the studies ranged from 18 to 49 years.

One of the most toxic classes of OCPs are cyclobenzenes and cyclohexanes substituted with six chlorine atoms. Hexachlorocyclohexane (HCH) occurs in four conformers: α, β, γ, and δ. Two reports showed the positive associations between HCH exposure with endometriosis among American women with disease confirmed by pelvic magnetic resonance imaging (Buck Louis et al. 2012) and by surgeon diagnosis (Upson et al. 2013). In the first study, Buck Louis et al. 2012 et al. in 2012 year found increasing odds of unreported earlier isomers of HCH: β-hexachlorocyclohexane in serum (*OR*_*A*djusted_ = 1.72; 95% *CI*: 1.09, 2.72) and γ-hexachlorocyclohexane in fat (*OR*_Adjusted_ = 1.27; 95% *CI*: 1.01, 1.59). One year later, Upson et al. confirmed these results in serum (*OR*_Adjusted_ = 1.3; 95% *CI*: 0.8, 2.4) but only for β conformer (Upson et al. 2013). The authors compared the relationship between exposure to OCPs and cases with all types of endometriosis. They also extracted and compared ovarian endometriosis cases. The association was stronger in analyses limiting cases to those with ovarian endometriosis (third vs. lowest quartile: *OR*_Adjusted_ = 2.5; 95% *CI*: 1.5, 5.2; highest vs. lowest quartile: *OR*_Adjusted_ = 2.5; 95% *CI*: 1.1, 5.3). Ploteau et al. (2016) determined the association between the internal exposure to chlorinated hydrocarbons and the presence of DIE in case–control study performed among 113 adult women enrolled during 2013–2015 in France (Ploteau et al. 2016). The association between the concentrations measured in omental versus parietal adipose tissue and between the concentrations determined in serum and parietal adipose tissue was significant for organochlorine pesticides. The positive associations between the presence of hexachlorobenzene (HCB) in adipose tissue and endometriosis risk have been found also 1 year later in another study by the same French authors (*OR*_Adjusted_ = 2.06; 95% *CI*: 1.2, 3.91) (Ploteau et al. 2017). Cases consisting of participants who had one of the stages of endometriosis (I, II, III, and IV) indicated an increased relationship among subgroups with ovarian endometriosis (*OR*). Another study indicating positive associations between β-HCH and ɣ-HCH and endometriosis was published by Pollack et al. in 2021 (Pollack et al. 2021). Application of adipose and serum ratio in this study with use Bayesian kernel machine regression (BKMR) allowed to calculate an increased odds for β-HCH and ɣ-HCH and endometriosis diagnosis in group of 339 women who had adipose and serum chemical measures (single chemical regression models: *OR*_ASR_ = 1.6; 95% *CI*: 1.2, 2.0) (Bobb et al. 2015; Pollack et al. 2021). Results not consistent with studies published before 2012 were described by Ploteau et al. (2017). The relationship between exposure to: dieldrin (*OR*_Adjusted_ = 2.72; 95% *CI*: 1.57, 5.11), trans-nonachlore (*OR*_Adjusted_ = 2.21; 95% *CI*: 1.24, 4.28), oxychlordane (*OR*_Adjusted_ = 3.22; 95% *CI*: 1.6, 7.7), cis-heptachlor (*OR*_Adjusted_ = 5.36; 95% *CI*: 2.44, 14.84), p,p-DDE (*OR*_Adjusted_ = 5.36; 95% *CI*: 2.44, 14.84), and endometriosis risk was found in adipose tissue among French population of 55 women with surgically confirmed DIE including 26 cases presented also OvE (control: 44). The negative effect of exposure to trans-nonachlor and development of endometriosis (*OR*_Adjusted_: 3.38; 95% *CI*: 2.06, 5.98) was confirmed also by Matta et al. in 2022 (Matta et al. 2022) in a preliminary case–control study conducted in France. Concentration of trans-nonachlor was measured in a serum in 186 women (cases: 56 with surgically confirmed endometriosis and controls: 130). Only one study assessed the relationship between past exposure to mirex — one of the most persistent and stable pesticide and occurrence of the endometriosis. Increasing odds, but not statistically significant, were found in the study performed among US population of 786 women recruited from WREN study (Women’s Risk of Endometriosis) with laparoscopic controls and surgically confirmed endometriosis (*OR*_Adjusted_ = 1.5; 95% *CI*: 1.0, 2.2) (Upson et al. 2013).

In conclusion, all of the studies found the association between exposure to organochlorines, at least one of the examined organochlorines, and endometriosis. Significant, positive relationship between nine organochlorine pesticides (β-hexachloro-cyklohexane, γ-hexachlorocyclohexane, HCB, dieldrin, trans-nonachlor, oxychlordane, cis-heptachlor, p,p-DDE and mirex) and endometriosis risk was exhibited.

### Dioxin and dioxin-like compounds

Dioxin and dioxin-like compounds are ubiquitous, very resistant environmental contaminants (Soave et al. 2015). They are mostly residual material of various industrial processes (waste incineration and iron/steel industries). They are chemically stable and readily dissolve in lipids. Dioxins are polycyclic aromatic hydrocarbons substituted by chlorine atoms. They are assigned to different branches due to the position of halogens. Dioxins and dioxin-like compounds include some polychlorinated biphenyls (PCBs), polychlorinated dibenzofurans (PCDFs), and polychlorinated dibenzo-p-dioxins (PCDDs).

#### Exposure to polychlorinated biphenyls

Polychlorinated biphenyls (PCBs) include dioxin-like polychlorinated biphenyls: DL-PCBs and non-dioxin-like polychlorinated biphenyls: NDL-PCBs. PCBs family has 209 possible congeners because biphenyl structure contains 10 positions to be substituted by chlorine atoms in different ways. Their toxicity and ability to accumulate in the body is strongly involved with structural properties and numbers of halogens (Miyawaki et al. 2015). Like most lipophilic contaminants, polychlorinated biphenyls do not easily degrade in the environment and readily bioaccumulate in food chains, adipose tissue (fat), and blood (Montano et al. 2022). Because of their ubiquity and resistance to environmental degradation, polychlorinated biphenyls began to be frequently studied for potential consequences for reproductive human health in recent several decades (Yao et al. 2017). The polychlorinated biphenyls disrupt the endocrine system by binding to estrogen or androgen receptors (Smarr et al. 2016).

The relationship between exposure to PCBs and endometriosis has been extensively studied in the past 30 years7 but still remains controversial due to inconsistent results. In this review, eight studies have been presented with results devoted to the relationship of endometriosis and exposure to polychlorinated biphenyls (Buck Louis et al. 2012; Ploteau et al. 2016; Pollack et al. 2021; Martínez-Zamora et al. 2015; Vichi et al. 2012; Kim et al. 2020; Roy et al. 2012; Neblett et al. 2020). The details of these studies are provided in the Supplementary Materials (Table S10). The half of the research was carried out in the USA — California, Baltimore, Georgia, Maryland (Buck Louis et al. 2012; Pollack et al. 2021; Roy et al. 2012; Neblett et al. 2020); the others: in Spain (Martinez Zamora et al. 2015), Italy (Vichi et al. 2012), and Korea (Kim et al. 2020) and in France (Ploteau et al. 2016). Most of them were designed as case–control study (Ploteau et al. 2016; Martínez-Zamora et al. 2015; Vichi et al. 2012; Kim et al. 2020), two as cohort study (Roy et al. 2012; Neblett et al. 2020), and two as case-sectional study (Buck Louis et al. 2012; Pollack et al. 2021). Endometriosis was confirmed by laparoscopic surgery and visualization (Ploteau et al. 2016; Pollack et al. 2021; Kim et al. 2020; Roy et al. 2012) or additionally by MRI in two studies (Buck Louis et al. 2012; Martínez-Zamora et al. 2015) and by a detailed questionnaire in one study (Neblett et al. 2020). The age of women participating in the studies ranged from 18 to 59 years. Volunteers were majorly recruited in hospitals and medical centers (Buck Louis et al. 2012; Pollack et al. 2021; Martínez-Zamora et al. 2015; Kim et al. 2020; Roy et al. 2012;) as well as one at the university (Vichi et al. 2012). Neblett et al. (2020) described a study group from the PBB Registry of Michigan Department of Community Health (MDCH)—women who worked at the chemical plant that produced PBB and their families.

Only one study published by Neblett et al. (2020) did not find an association between none of PCB and endometriosis. Most of reported congeners of polychlorinated biphenyls like PCB 114, 118, 126, 136, 138, 153, 170, 180, and 189 reported in two studies indicate strong associations with occurrence of endometriosis *OR*_Adjusted_ = 1.62; 95% *CI*: 1.21, 2.17 for 3,3′,4,4′,5-pentachlorobiphenyl (PCB 126) (Martínez-Zamora et al. 2015) and *OR*_Adjusted_ = 3.97; 95% *CI*: 1.51, 10.5 (for medium level 63–104 ng/g fat) for 2,2′,4,4′,5,5′-hexachlorobiphenyl (PCB 153) (Vichi et al. 2012).

In the study published by Sofo et al. in 2015, one of the most toxic dioxin-like congener — 2,3,7,8-tetrachlorodibenzo-p-dioxin (TCDD), was significantly associated with endometriosis risk (Sofo et al. 2015). Twelve coplanar structures from 209 congeners of polychlorinated biphenyls including: 4 non-ortho PCBs (PCB 77, 81,126 and 169) and 8 mono-ortho PCBs (PCB 105,114,118,123,156, 157, 167, and 189) are called dioxin-like PCBs (DL-PCBs) and may exhibit similar biological, high toxicity like TCDD (Fernandez-Salguero et al. 1996). Five coplanar PCB congeners (114, 118, 126, 156, and 189) were described in studies above (Martínez-Zamora et al. 2015; Roy et al. 2012; Vichi et al. 2012) with increasing odds belonging to group dioxin-like compounds, with the absence of chlorine in positions 2, 2′, 6, and 6′. Probably they can be competitive to dioxins, bind the ligand-activated nuclear transcription factor — aryl hydrocarbon receptor (AhR) (Obaid 2000, and what is crucial in disrupting the endocrine system. Decreasing odds ratio for congener 156 described Buck Louis et al. (2012) at population of 473 American women in omental fat (*OR*_Adjusted_ = 0.74; 95% *CI*: 0.57, 0.96). In case of congeners PCB 28 — three substituted chlorine atoms with lower odds was also observed (*OR*_Crude_ = 1.30; 95% *CI*: 1.04, 1.62 but *OR*_Adjusted_ = 1.16; 95% *CI*: 0.92, 1.47). For congener PCB 52 (Kim et al. 2020) and PCB 74 (Buck Louis et al. 2012) — with four substituted chlorine atoms also with decreasing odds ratio was found (*OR*_Adjusted_ = 0.350, 95% *CI*: 0.138, 0.885 — higher tertile with the lowest; *OR*_Adjusted_ = 0.72; 95% *CI*: 0.55, 0.93). Below four chlorine atoms in structure of PCB reported data usually shows greater odds of association with the exposure and endometriosis for example: PCB 114 — five chlorine atoms (*OR*_Adjusted_ = 2.47; 95% *CI*: 1.24, 5.64 (Martínez-Zamora et al. 2015) and *OR*_Adjusted_ = 3.01; 95% *CI*: 2.25, 3.77 (Roy et al. 2012)) PCB 136 — six chlorine atoms (*OR*_Adjusted_ = 1.79; 95% *CI*: 1.03, 2.55) (Roy et al. 2012), PCB 170 and 180 — seven chlorine atoms (high > 60.4 ng/g fat: *OR*_Adjusted_ = 2.94; 95% *CI*: 1.14, 7.57, and *OR*_Adjusted_ = 3.13; 95% *CI*: 1.28, 7.68) (Buck Louis et al. 2012). In the case of more substituted congeners, two studies reported association between molecules with the presence eight — PCB-201 and nine chlorine atoms PCB-206 and endometriosis (Buck Louis et al. 2012; Pollack et al. 2021)]. The lower substituted polychlorinated biphenyl PCB-201 indicates only crude increasing odds (*OR*_Crude_ = 1.28; 95% *CI*: 1.03, 1.60), but in the same group of women and identical parameters of case-sectional study, nonachlorobiphenyl (PCB-206) showed decreasing odds of association between the exposure to polychlorinated biphenyls and endometriosis (*OR*_Adjusted_ = 0.79; 95% *CI*: 0.65, 0.97) (Buck Louis et al. 2012). Whereas, in the study conducted by Pollack et al. (2021) among 339 American women with surgically confirmed endometriosis, mixtures of adipose and ASR estrogenic PCBs (congener 49 and 201) were associated with incident endometriosis diagnosis. Vichi et al. (2012) and Kim et al. (2020) obtained in two independent studies inconsistent results for congener PCB-118. The first author determined pentachlorobiphenyl serum concentrations from 63 cases and 63 controls, with laparoscopic diagnosis and histologic confirmation of endometriosis. Significant relationship with PCB 118 and occurrence of endometriosis was observed (high: *OR*_Adjusted_ = 3.18; 95% *CI*: 1.26, 8.01, medium high: *OR*_Adjusted_ = 2.62; 95% *CI*: 1.18, 5.83 — versus low level (first tertile) (Vichi et al. 2012). On the other hand, in case–control study 2,3′,4,4′,5-pentachlorobiphenyl plasma concentration in 61 cases (surgical and histological evidence of advanced endometriosis) was lower in comparison to 99 Asian control women (*OR*_Adjusted_ = 0.320; 95% *CI*: 0.125, 0.817 — comparison the highest tertile levels with the lowest tertile levels) (Kim et al. 2020). Ploteau et al. (2016) have investigated distribution of persistent organic pollutants in serum, omental and parietal adipose tissues, and their correlation with endometriosis. They determined the concentration of six PCBs (PCBs-28, 52, 101, 138, 153, and 180) in a group of 113 adult women with surgically confirmed endometriosis. The authors found that the correlation between both kinds of adipose tissue was strongly significant for analyzed polychlorinated biphenyls with equivalence of the measures performed in both tissues. No statistically significant relationship between polychlorinated biphenyls (total: congeners 118, 138, 153, and 180) and development of endometriosis was found in one study performed by Neblett et al. (2020). In the study among women (18–59 years) with endometriosis diagnosed by a doctor, who lived in the state of Michigan during the time of PCB contamination (1973–1974), the total concentration of four PCBs (PCB — congeners: 118, 138, 153, and 180) were measured and no statistically significant relationship between exposure to these PCBs and endometriosis was observed (*OR*_Adjusted_ = 1.02; 95% *CI*: 0.68, 1.53).

Most of the presented studies on the exposure to polychlorinated biphenyls found a relationship between endometriosis and PCBs, at least one of the examined polychlorinated derivatives. Vichi et al. (2012) and Kim et al. (2020) presented opposite results for congener PCB 118 in two independent studies. This inconsistency in the value of odds ratio may be due to the differences in the selection of study groups, type of compound, and nature of biological fluids in which concentrations were measured. However, most of these human studies, mentioned above, showed a significant positive association (seventeen PCB in eight studies) and only one PCB (congener 206) and one study conducted by Neblett et al. (2020) did not show any relationship between polychlorinated biphenyl concentration in biological matrices and endometriosis.

#### Exposure to polychlorinated dibenzodioxins and polychlorinated dibenzofurans

Group of polychlorinated biphenyls consists of polychlorinated dibenzodioxins (there are 75 PCDDs) and polychlorinated dibenzofurans (there are 135 PCDFs) and both compose dioxin-like compounds. Responsible for most toxic effects of these chemicals is the ability to bind a specific aryl hydrogen receptor (AhR). They accumulate in tissues with high fat content and the level of polychlorinated biphenyls increases with age in humans (Ozga-Stachurska et al. 2022).

Two studies on the exposure to polychlorinated dibenzodioxins and polychlorinated dibenzofurans and endometriosis were performed in the last 10 years (Ploteau et al. 2017; Martínez-Zamora et al. 2015). The details of these studies are provided in the Supplementary Materials (Table S11). One of the studies was conducted in Spain and endometriosis was confirmed by laparoscopic surgery, magnetic resonance imaging (MRI) and transvaginal sonography (Martínez-Zamora et al. 2015). In a second study published in France, the women had surgical confirmation of endometriosis (Ploteau et al. 2017). Both studies had case–control design. The age of recruited women was in the range of 18–45 years. Volunteers were recruited from a university hospital located in Spain (Martínez-Zamora et al. 2015) or from population-based on clinical case–control study conducted in France (Ploteau et al. 2017). The exposure was assessed on two dibenzodioxins: 2,3,7,8-tetrachlorodibenzodioxin and 1,2,3,7,8-pentachlorodibenzodioxin (1,2,3,7,8 — PeCDD and 2,3,7,8 — TCDD) and two dibenzofurans: 2,3,4,7,8-pentachlorodibenzofuran and octochlorodibenzofuran (2,3,4,7,8 — PeCDF and OCDF). The exposure was analyzed in adipose tissues. The authors obtained similar results for dibenzodioxins. Both 1,2,3,7,8 — PeCDD (*OR*_Adjusted_ = 1.82; 95% *CI*: 1.36, 7.14 (Martínez-Zamora et al. 2015), *OR*_Adjusted_ = 2.20; 95% *CI*: 1.23, 4.25 (Ploteau et al. 2017)) and 2,3,7,8 — TCDD (*OR*_Adjusted_ = 1.41; 95% *CI*: 1.12, 2.10 (Martínez-Zamora et al. 2015), *OR*_Adjusted_ = 1.65; 95% *CI*: 0.95, 3.02 (Ploteau et al. 2017)) were significantly associated with endometriosis. Additionally, in case of 2,3,4,7,8-pentachlorodibenzofuran (2,3,4,7,8 — PeCDF) from PCDF class, both studies reported that exposure to this compound may disturb the endocrine system and cause development of endometriosis (*OR*_Adjusted_ = 1.94; 95% *CI*: 1.27, 5.16 (Martínez-Zamora et al. 2015), *OR*_Adjusted_ = 2.21; 95% *CI*: 1.06, 5.16 (Ploteau et al. 2017)). However, in the case of octochlorodibenzofuran from polychlorinated dibenzofuran class, the results of the studies performed in Spain and France are not consistent. Ploteau et al. (2017) found a significant association *OR*_Adjusted_ = 5.42; 95% *CI*: 2.73, 12.85, whereas Martinez et al. (2015) did not find association between octochlorodibenzofuran and endometriosis. The association with endometriosis, among detected polychlorinated dibenzofurans, indicates that OCDF has moderate agonist activity at the aryl hydrogen receptor. These results suggest that other mechanisms may have a role on the pathogenesis of endometriosis for example POP-induced immunotoxic effects (Egsmose et al. 2016) and/or gene polymorphisms (Vichi et al. 2012). In conclusion, only two studies assess the relationship between exposure to polychlorinated dibenzodioxins and polychlorinated dibenzofurans, and both found an increased risk of endometriosis related to such type of exposure.

### Polybrominated biphenyls

Polybrominated biphenyls (PBBs) are a class of halogenated biphenyls where 1–10 hydrogen atoms are substituted with bromine atoms. PBBs have 209 possible individual congeners and the same substitutions configuration by bromine atoms as polychlorinated biphenyls. Polybrominated biphenyls (13 milion pounds production by 6 years in the USA) have been used as flame retardants in textiles, electronic equipment, and plastics and still occur in air, dust, soil, seafood (mainly fish), milk, meat, and dairy products (Jagić et al. 2021).

In the previous decade, the association between endometriosis and polybrominated biphenyls was investigated in three studies (Ploteau et al. 2017; Neblett et al. 2020; Gerkowicz et al. 2020). The details of these studies are provided in the Supplementary Materials (Table S12). Each of the studies described inconsistent results in the field of endometriosis risk and exposure to PBBs — in one study with increasing odds ratio (Ploteau et al. 2017), in the second study with decreasing odds ratio (Gerkowicz et al. 2020) and the last with no relationship between endometriosis and polybrominated biphenyls (Neblett et al. 2020). In all the three studies, only four congeners of PBBs were statistically significant (Ploteau et al. 2017; Gerkowicz et al. 2020). Two of the cross-sectional studies were conducted in the USA (Georgia), where endometriosis was confirmed by a detailed questionnaire (Neblett et al. 2020, Gerkowicz et al. 2020). In one case–control study published in France, the women had surgical confirmation of endometriosis (Ploteau et al. 2017). The age of recruited women was in the range of 18–59 years. Neblett et al. (2020) and Neblett et al. (2020) selected participants from the Michigan Polybrominated Biphenyl (PBB) Registry — included people involved with farms affected by PBB (Neblett et al. 2020; Gerkowicz et al. 2020). Ploteau et al. (2017) data were collected as part of a clinical case–control study of endometriosis between 2013 and 2015 on a French population. The increasing odds with exposure to polybrominated biphenyls and endometriosis risk was found only in the case of congener PBB 153 (2,2′,4,4′,5,5′-hexabromobiphenyl) (*OR*_Adjusted_ = 3.91; 95% *CI*: 1.60, 11.60) (Ploteau et al. 2017) performed among 99 women (55 with surgical diagnosis of DIE in adipose tissue). On the other hand, Gerkowicz et al. found decreasing odds for the same congener and for three other derivatives: PBB-77 (3,3′,4,4′-tetrabromobiphenyl), PBB-101 (2,2′,4,5,5′-pentabromobiphenyl) and PBB-180 (2,2′,3,3′,4,4′,5-heptabromobiphenyl) (Gerkowicz et al. 2020). In this study, an analysis of the total (four congeners) polybrominated biphenyls levels in serum was carried among 305 women, 65 with endometriosis confirmed by a detailed questionnaire (*OR*_Adjusted_ = 0.97; 95% *CI*: 0.93, 0.99). In the same year, Neblett et al. (2020) investigated association between the same four congeners (PBB-77, PBB-101, PBB-153, and PBB-180) of polybrominated biphenyls and endometriosis. Despite similar conditions of studies (women from Michigan, cross-sectional study, PBB serum concentration, and endometriosis confirmed by detailed questionnaire), they did not find a relationship between endometriosis and selected PBBs. Due to inconsistent results performed, further research and surveillance are needed to determine if and how PCB and PBB may impact on endometriosis.

### Polybrominated diphenyl ethers

Polybrominated diphenyl ethers (PBDEs) are one class of flame retardant chemicals (brominated flame retardants — BFRs) that are added to a range of products (textiles and thermoplastics used in electronics) to make them resistant to burning. Two phenyls rings coupled with oxygen are able to bind from one to ten bromine atoms which give opportunity to make 209 congeners of PBDEs (Pietroń and Małagocki 2017). They are very stable in the environment, indicate high lipophilicity and hydrophobicity, and tend to travel for a long distance with air and water as well as bioaccumulate in human tissues. Exposure to these persistent organic pollutants can take place by respiratory system, oral ingestion, breastfeeding, or skin absorption (Ohoro et al. 2021).

The association between polybrominated diphenyl ethers and endometriosis has been studied in three studies during the last 10 years (Buck Louis et al. 2012; Ploteau et al. 2017; Pollack et al. 2021). The details of these studies are provided in the Supplementary Materials (Table S13). Ploteau et al. (2017) and Pollack et al (2021) described case–control studies among the French population of women enrolled in 2013–2015 years as population-based studies (99 women) and between 2007 and 2009 recruited from the surgical centers’ catchment areas (473 women), respectively. The age of participants was in the range of 18–45 years. In two studies, endometriosis was confirmed by surgical visualization (Ploteau et al. 2017; Pollack et al. 2021), and in one study, histologic confirmation and magnetic resonance imaging were used (Buck Louis et al. 2012). In three presented researches, only one study showed the significant increasing odds in development of endometriosis for one endocrine disruptor substance from a group of PBDEs (Ploteau et al. 2017). This study was conducted in France on a group of 99 women including 55 cases with deep infiltrating endometriosis and 26 cases from whom additionally ovarian endometriosis was confirmed. Levels of eight polybrominated diphenyl ethers (PBDE-28, 47, 99, 100, 153, 154, 183, and 209) were measured in adipose tissues. Only congener with six bromine atoms PBDE-183 (2,2′,3,4,4′,5′,6-heptabromodiphenyl ether) indicated positive association with endometriosis risk for group with deep infiltrating endometriosis (DIE: *OR*_Adjusted_ = 1.64; 95% *CI*: 1.05, 2.71). The authors did not find significant results in comparison cases with DIE and OvE ( DIE + OvE: *OR*_Adjusted_ = 1.29 (95% *CI*: 0.76, 2.30). The same compounds, except congener PBB-28, was analyzed at US population in a study among 473 women from operative cohort and 127 women from the population cohort (204 cases with endometriosis) in another study by Buck Louis et al. (2012). Concentration of seven polybrominated diphenyl ethers have been measured (PBDE-47, 99, 100, 153, 154, 183, and 209) in omental fat. Despite higher omental fat level of PBDE-183 in women with surgically confirmed EM, the authors did not find a significantly increasing odds ratio in endometriosis. However, congener PBDE-47 (2,2′,4,4′-tetrabromodiphenyl ether) indicated decreasing odds in development of endometriosis in the operative cohort (*OR*_Adjusted_ = 0.70; 95% *CI*: 0.55, 0.90). The third study published in the year 2021 by Pollack et al. has investigated adipose to serum ratio (ASR) total concentration of six PBDEs (PBDE-47, 99, 100, 153, 154, and 209) in group of 190 American women and 283 controls with surgically confirmed endometriosis. Median polybrominated biphenyl ether ASRs were mostly higher among women without than with endometriosis, except for PBDE-154 and PBDE-209. The authors did not find that the adipose to serum ratio for polybrominated diphenyl ethers was associated with endometriosis. These various results of presented research may indicate that PBDEs are metabolized to some extent which points to lacking association.

### Per- and polyfluoroalkyl substances

After the first synthesis of PFASs in 1930s (first used in the 1940s), polyfluoroalkyl derivatives are widely used in the industry as surfactants, lubricants, floor waxes, fire-fighting agents, denture cleaners, shampoos, pharmaceutical products, and in food packaging (OECD 2018). These organofluorine chemical compounds with multiple fluorine atoms attached to an alkyl chain are present in the environment in the form of dissociated anions and are extremely resistant in the environment (Vierke et al. 2013). Above 1400 individual PFAS have been introduced for industry (Evich et al. 2022). PFASs easily accumulate in the ocean and marine organism food chains due to their anionic properties. Human exposure occurs through contaminated food, drinking water, and house dust. PFASs have not been categorized officially as endocrine disruptor chemicals yet (Kato et al. 2015; Kranthi Kumar et al. 2017). They are considered as endocrine-toxic due to their ability to changing thyroid hormone level and impropering functioning of the reproductive system by disorders in activity of hypothalamic–pituitary–gonadal (HPA) axis in animals exposed to PFOS (Zhao et al. 2010; Luebker et al. 2015). PFASs including two groups of compounds: PFCAs — perfluoroalkyl carboxylic acids (this group contains following acids: PFOA-perfluorooctanoic acid, PFHxA-perfluorohexanoic acid, PFNA — perfluorononanoic acid and PFDA — perfluorodecanoic acid) and PFSAs — perfluoroalkane sulfonic acids (this group contains following acids: PFOS — perfluorooctane sulfonic acid, PFHxS — perfluorohexane sulfonic acid and PFBS — perfluorobutane sulfonic acid).

Exposure to per- and polyfluoroalkyl substances are ubiquitous and interest in this topic is still growing. Five studies investigated the association between PFASs and endometriosis in the last 10 years (Matta et al. 2022; Louis et al. 2012; Campbell et al. 2016; Wang et al. 2017; Hammarstrand et al. 2021). The details of these studies are provided in the Supplementary Materials (Table S14). Three studies using case–control study design (Louis et al. 2012; Campbell et al. 2016; Wang et al. 2017), one study had a clinical-based approach (Matta et al. 2022), and one study was population-based comparison (Hammarstrand et al. 2021). Two studies were performed in the USA (California, Utah) (Louis et al. 2012; Campbell et al. 2016), one in China (Wang et al. 2017), one in Swedish (Hammarstrand et al. 2021), and one in France (Matta et al. 2022). Endometriosis was confirmed by surgically visualization in operative and by MRI in population in a study performed by Louis et al. (2012); in another study confirmation was self-reported based on doctor diagnosis (Campbell et al. 2016), and in two studies surgically confirmation was used (Wang et al. 2017; Matta et al. 2022). In a study conducted in Swedish, endometriosis was confirmed by ICD codes (International Classification of Diseases) (Hammarstrand et al. 2021). Volunteers were in the range of 18–50 years, and the most were enrolled in hospitals and clinical centers (Matta et al. 2022; Louis et al. 2012; Wang et al. 2017). Hammarstrand et al. (2021) conducted a population-based comparison between women from areas with water supply from the highly contaminated waterworks, non-exposed women, and occurrences of endometriosis. One study combined data from the 2003–2004 and 2005–2006 National Health and Nutrition Examination Survey (NHANES) cycles (Louis et al. 2012). Three studies found association between endometriosis and exposure to PFASs (Louis et al. 2012; Louis et al. 2012; Wang et al. 2017) including four compounds from a group of PFASs which showed increasing odds with endometriosis risk. The results of the studies performed by Campbell et al. (2016) and Louis et al. (2012) indicated that three fluoroalkyl: PFOA, PFNA, and PFOS, may cause development of endometriosis. In the study conducted by Campbell et al. (2016), twelve per- and polyfluoroalkyl substances were detected in serum samples of 753 American women categorized into tertiles and quartiles. The results indicated that PFOA (*OR*_Adjusted_: 5.45; 95% 1.19; 25.04 — third tertile), PFNA (*OR*_Adjusted_: 5.27; 95% *CI*: 1.20, 23.06 — third tertile) and PFOS (*OR*_Adjusted_: 3.48; 95% *CI*: 1.00, 12.00 — fourth quartile) have exhibited significant association with endometriosis risk. In a second study conducted by Louis et al. (2012), nine PFASs were analyzed serum among the operative cohort — 495 women and population cohort — 131 women. In this study, two organofluorine substances: PFOA (*OR*_Adjusted_: 1.89; 95% *CI*: 1.17, 3.06) and PFNA (*OR*_Adjusted_: 2.20; 95% *CI*: 1.02, 4.75) have indicated increasing odds with development of endometriosis. A third study published by Wang et al. in (2017) investigated 157 individuals with surgically confirmed endometriosis and 178 controls. The authors have found that only perfluorobutane sulfonic acid (PFBS) was statistically significantly associated with endometriosis risk among ten measured and analyzed PFASs.

Two studies reported by Matta et al. (2022) and Hammarstrand et al. (2021) did not show association between endometriosis and per- and polyfluoroalkyl substances. They together have determined 19 PFASs, but no increased hazard ratio (HR) in the first Hammarstrand’s study and no relevant association in the Matta’s study have been shown. Hammarstrand et al. (2021) did not measure the PFAS levels in biological fluid. In this study, the authors investigated the possible associations between PFAS exposure and endometriosis in a cohort exposed to PFAS through drinking water contaminated by firefighting foams used at a nearby airfield. In conclusion, the three studies (Louis et al. 2012; Campbell et al. 2016; Wang et al. 2017) published in recent 10 years have shown that exposure to PFASs has a crucial contribution in development of endometriosis. However, the European Food Safety Authority (EFSA) concluded in 2018 that there was not yet enough evidence to establish a clear connection between PFAS exposure and endometriosis (Knutsen et al. 2018).

## Discussion

Most of the studies reviewed in this paper showed an association between exposure persistent EDCs and endometriosis. All reviewed studies are shown in Table 1. Only two studies did not find associations with endometriosis and analyzed persistent endocrine disruptor chemicals. The remaining studies (21) indicated relationship between endometriosis and exposure to metals, organochlorine pesticides, polychlorinated biphenyls, polybrominated biphenyls, polychlorinated dibenzo-p-dioxins, polychlorinated dibenzofurans, polybrominated diphenyl ethers, and per- and polyfluoroalkyl substances. Seven studies included information about exposure to metals or trace elements and occurrence of endometriosis (Zhang et al. 2021; Lai et al. 2017; Tanrikut et al. 2014; Pollack et al. 2013; Kim et al. 2021; Silva et al. 2013, Yılmaz et al. 2020). In recent 10 years, 21 metals have been analyzed and reported in correlation between exposure and occurrence of endometriosis. Six of them: cadmium, copper, lead, mercury, zinc, and chromium indicated positive association with endometriosis risk. Increasing odds ratio was found in six studies. Additionally for cadmium and zinc, a decreasing odds ratio was found (Lai et al. 2017; Pollack et al. 2013). In case of 15 metals, no associations was found (nickel, iron, manganese, aluminium, antimony, arsenic, barium, beryllium, cesium, cobalt, molybdenum, tellurium, thallium, tin, and tungsten). All nine organochlorine pesticides described in six studies (Buck Louis et al. 2012; Upson et al. 2013; Ploteau et al. 2016, 2017; Pollack et al. 2021; Matta et al. 2022) have positive effects on development endometriosis (β-hexachlorocyklohexane, γ-hexachlorocyclohexane, HCB, dieldrin, trans-nonachlor, oxychlordane, cis-heptachlor, p,p-DDE, and mirex). In each case, an increasing odds ratio was observed. Association between endometriosis and polychlorinated biphenyls was investigated in eight studies (Buck Louis et al. 2012; Ploteau et al. 2016; Pollack et al. 2021; Martínez-Zamora et al. 2015; Vichi et al. 2012; Kim et al. 2020; Roy et al. 2012; Neblett et al. 2020) including 18 congeners of PCBs (PCB-28, 49, 52, 74, 101, 114, 118, 126, 136, 138, 151, 153, 156, 170, 180, 189, 201, and 206). Almost all the authors found increasing odds ratio for at least one compound despite Neblett et al. (2020). For congeners, PCB-52 (Ploteau et al. 2016; Kim et al. 2020), PCB-114 (Martínez-Zamora et al. 2015; Roy et al. 2012), PCB-118 (Vichi et al. 2012; Kim et al. 2020), and PCB-156 (Buck Louis et al. 2012; Martínez-Zamora et al. 2015) differences in statistically significant value of odds ratio (increasing and decreasing) were presented. Only two studies describe exposure to polychlorinated dibenzo-p-dioxins and dibenzofurans (Ploteau et al. 2017, Martínez-Zamora et al. 2015). Four derivatives: 2,3,7,8 — TCDD, 1,2,3,7,8 — PeCDD, 2,3,4,7,8 — PeCDF, and OCDF indicate increased odds of endometriosis. Four polybrominated biphenyls like PBB-77, 101, 153, and 180 were related to endometriosis (Gerkowicz et al. 2020). On the other hand, Neblett et al. (2020) did not find association with occurrence of endometriosis and all four PBBs. Ploteau et al. (2017) showed increasing odds ratio in endometriosis only for congener PBB-153. Three studies include exposure to polybrominated diphenyl ethers (Buck Louis et al. 2012; Ploteau et al. 2017, Pollack et al. 2021). PBDE-47, 99, 100, 153, 154, 183, and 209 were found with increased odds in development of endometriosis. For congener, PBDE-47 was also shown decreasing odds ratio by Buck Louis et al. (2012) opposite to results obtained by Pollack et al. (2021). In the case of PBDE-183, Buck Louis et al. (2012) did not find relationship with endometriosis. Thirteen per- and polyfluoroalkyl substances (PFPeA, PFHxA, PFHpA, PFOA, PFNA, PFDA, PFUnDA, PFDoDA, PFBS, PFHxS, PFHpS, PFOS, and PFUnA) have been investigated in relation to endometriosis in five studies (Matta et al. 2022; Louis et al. 2012; Campbell et al. 2016; Wang et al. 2017; Hammarstrand et al. 2021), but only three of them increase odds ratio in development of endometriosis (Louis et al. 2012; Campbell et al. 2016; Wang et al. 2017). Matta et al. (2022) and Hammarstrand et al. (2021) did not find association between endometriosis and analyzed fluoroalkyl derivatives.

**Table 1:** The association between exposures to selected persistent endocrine disrupting chemicals and endometriosis

No	Class of EDCs/authors of study	Associations with endometriosis ( +) positive effect	Associations with endometriosis ( −) adverse effect	No associations with endometriosis
Metals (7 studies)				
1	Pollack et al. (2013)	( +) Chromium ( +) Copper	( −) Cadmium	Antimony, arsenic, barium, beryllium, cesium, cobalt, lead, manganese, mercury, molybdenum, nickel, tellurium, thallium, tin, tungsten, zinc
2	Silva et al. (2013)	( +) Lead		Cadmium, nickel
3	Tanrikut et al. (2014)	( +) Cadmium		Lead, mercury
4	Lai et al. (2017)		( −) Zinc	Cadmium, chromium, copper, iron, lead, manganese, mercury
5	Yılmaz et al. (2020)	( +) Zinc		Aluminum, copper, nickel
6	Kim et al. (2021)	( +) Lead		Cadmium
7	Zhang et al. (2021)	( +) Mercury		Cadmium, lead
Organochlorine pestiicides — OCPs (6 studies)				
1	Buck Louis et al. 2012 et al. (2012)	( +) β -hexachlorocyklohexane (HCH) ( +) γ- hexachlorocyclohexane		
2	Upson et al. (2013)	( +) β -hexachlor-cyklohexane (HCH) ( +) mirex		
3	Ploteau et al. (2016)	( +) β -hexachlorocyklohexane (HCH) ( +) γ- hexachlorocyclohexane ( +) Cis-heptachlor ( +) Dichlorodiphenyldichloroethylene (p,p-DDE) ( +) Hexachlorobenzene (HCB) ( +) Oxychlordane ( +) Trans-nonachlor		
4	Ploteau et al. (2017)	( +) β-hexachlorocyklohexane (HCH) ( +) Cis-heptachlor ( +) Dichlorodiphenyldichloroethylene (p,p-DDE) ( +) Dieldrin ( +) Hexachlorobenzene (HCB) ( +) Oxychlordane ( +) Trans-nonachlor		
5	Pollack et al. (2021)	( +) β -hexachlorocyklohexane (HCH) ( +) γ- hexachlorocyclohexane		
6	Matta et al. (2022)	( +) Trans-nonachlor		
Polychlorinated biphenyls (8 studies)				
1	Buck Louis et al. 2012 et al. (2012)	( +) PCB 28 ( 2,4,4'-trichlorobiphenyl) ( +) PCB 151 (2,2',3,5,5',6-hexachlorobiphenyl) ( +) PCB 201 (2,2',3,3',4,5',6,6'-octachlorobiphenyl)	( −) PCB 74 (2,4,4',5-Tetrachlorobiphenyl) ( −) PCB 156 (2,3,3',4,4',5-Hexachlorobiphenyl) ( −) PCB 206 (2,2',3,3',4,4',5,5',6-Nonachlorobiphenyl)	
2	Roy et al. (2012)	( +) PCB 114 (2,3,4,4',5-pentachlorobiphenyl) ( +) PCB 136 (2,2',3,3',6,6'-hexachlorobiphenyl)		
3	Vichi et al. (2012)	( +) PCB 118 (2,3',4,4',5-pentachlorobiphenyl) ( +) PCB 138 (2,2',3,4,4',5'-hexachlorobiphenyl) ( +) PCB 153 (2,2',4,4',5,5'-hexachlorobiphenyl) ( +) PCB 170 (2,2',3,3',4,4',5-heptachlorobiphenyl) ( +) PCB 180 (2,2',3,4,4',5,5'-heptachlorobiphenyl)		
4	Martinez-Zamora et al. (2015)	( +) PCB 114 (2,3,4,4',5-pentachlorobiphenyl) ( +) PCB 126 (3,3',4,4',5-pentachlorobiphenyl) ( +) PCB 156 (2,3,3',4,4',5-hexachlorobiphenyl) ( +) PCB 189 (2,3,3',4,4',5,5'-heptachlorobiphenyl)		
5	Ploteau et al. (2016)	( +) PCB 28 ( 2,4,4'-trichlorobiphenyl) ( +) PCB 52 (2,2',5,5'-tetrachlorobiphenyl) ( +) PCB 101 (2,2',4,5,5'-pentachlorobiphenyl) ( +) PCB 138 (2,2',3,4,4',5'-hexachlorobiphenyl) ( +) PCB 153 (2,2',4,4',5,5'-hexachlorobiphenyl) ( +) PCB 180 (2,2',3,4,4',5,5'-heptachlorobiphenyl)		
6	Kim et al. (2020)		( −) PCB 52 (2,2',5,5'-tetrachlorobiphenyl) ( −) PCB 118 (2,3',4,4',5-pentachlorobiphenyl)	
7	Neblett et al. (2020)			PCB 118 (2,3',4,4',5-pentachlorobiphenyl) PCB 138 (2,2',3,4,4',5'-hexachlorobiphenyl) PCB 153 (2,2',4,4',5,5'-hexachlorobiphenyl) PCB 180 (2,2',3,4,4',5,5'-heptachlorobiphenyl)
8	Pollack et al. (2021)	( +) PCB 201 (2,2',3,3',4,5',6,6'-octachlorobiphenyl) ( +) PCB 49 (2,2',4,5'-tetrachlorobiphenyl)		
Polychlorinated dibenzo-p-dioxins and polychlorinated dibenzofurans (2 studies)				
1	Martinez-Zamora et al. (2015)	( +) 1,2,3,7,8 – PeCDD (1,2,3,7,8-pentachloro-dibenzodioxin) ( +) 2,3,4,7,8 – PeCDF (2,3,4,7,8-pentachloro-dibenzofuran) ( +) 2,3,7,8 – TCDD (2,3,7,8-tetrachlorodibenzodioxin)		OCDF (octochlorodibenzofuran)
2	Ploteau et al. (2017)	( +) OCDF (octochlorodibenzofuran) ( +) 1,2,3,7,8 – PeCDD (1,2,3,7,8-pentachloro-dibenzodioxin) ( +) 2,3,4,7,8 – PeCDF (2,3,4,7,8-pentachloro-dibenzofuran)		2,3,7,8 – TCDD (2,3,7,8-tetrachlorodibenzodioxin)
Polybrominated biphenyls (3 studies)				
1	Ploteau et al. (2017)	( +) PBB 153 (2,2’,4,4’,5,5’-hexabromobiphenyl)		
2	Gerkowicz et al. (2020)		( −) PBB 77 (3,3’,4,4’-tetrabromobiphenyl) ( −) PBB 101 (2,2’,4,5,5’-pentabromobiphenyl) ( −) PBB 153 (2,2’,4,4’,5,5’-hexabromobiphenyl) ( −) PBB 180 (2,2’,3,3’,4,4’,5-heptabromobiphenyl)	
3	Neblett et al. (2020)			PBB 77 (3,3’,4,4’-tetrabromobiphenyl) PBB 101 (2,2’,4,5,5’-pentabromobiphenyl) PBB 153 (2,2’,4,4’,5,5’-hexabromobiphenyl) PBB 180 (2,2’,3,3’,4,4’,5-heptabromobiphenyl)
Polybrominated diphenyl ethers (3 studies)				
1	Buck Louis et al. 2012 et al. (2012)		( −) PBDE 47 (2,2’,4,4’-tetrabromodiphenyl ether)	PBDE 183 (2,2’,3,4,4’,5’,6-heptabromodiphenyl ether)
2	Ploteau et al. (2017)	( +) PBDE 183 (2,2’,3,4,4’,5’,6-heptabromodiphenyl ether)		
3	Pollack et al. (2021)	( +) PBDE 47 (2,2’,4,4’-tetrabromodiphenyl ether) ( +) PBDE 99 (2,2’,4,4’,5-pentabromodiphenyl ether) ( +) PBDE 100 (2,2’,4,4’,6-pentabromodiphenyl ether) ( +) PBDE 100 (2,2’,4,4’,6-pentabromodiphenyl ether) ( +) PBDE 153 (2,2’,4,4’,5,5’-hexabromodiphenyl ether) ( +) PBDE 154 (2,2’,4,4’,5,6’-hexabromodiphenyl ether) ( +) PBDE 209 (decabromodiphenyl ether)		
Per- and polyfluoroalkyl substances (5 studies)				
1	Louis et al. (2012)	( +) PFNA (perfluorononanoic acid) ( +) PFOA (Perfluorooctanoic acid)		
2	Campbell et al. (2016)	( +) PFNA (perfluorononanoic acid) ( +) PFOA (Perfluorooctanoic acid) ( +) PFOS (Perfluorooctane sulfonate)		PFHxS (perfluorohexane sulfonate)
3	Wang et al. (2017)	( +) PFBS (perfluorobutane sulfonic acid)		
4	Hammarstrand et al. (2021)			PFDA (perfluorodecanoic acid) PFDoDA (perfluorododecanoic acid) PFNA (perfluorononanoic acid) PFHpA ( perfluoroheptanoic acid) PFHxA (perfluorohexanoic acid) PFHxS (perfluorohexane sulfonate) PFHpS (perfluorohexane sulfonic acid) PFOA (perfluorooctanoic acid) PFOS (perfluorooctane sulfonate) PFPeA (perfluoropentanoic acid) PFUnDA (perfluoroundecanoic acid)
5	Matta et al. (2022)			PFDA (perfluorodecanoic acid) PFNA (perfluorononanoic acid) PFHpS (perfluorohexane sulfonic acid) PFOA (perfluorooctanoic acid) PFOS (perfluorooctane sulfonate) PFUnA (perfluoroundecanoic acid)

Despite many studies dedicated to endometriosis still there is no medication for this disease. Therefore, only early diagnosis of endometriosis can slow or inhibit its natural progression. Moreover, recent studies reported that the mammals are more susceptible to the effects of EDC, and studies of exposure to EDCS should be conducted during early life development and pregnancy (Le Magueresse-Battistoni Le et al. 2020; Stephens et al. 2022). Many academic institutions and organizations conduct research to identify effective models of endometriosis prevention, diagnosis, treatment, and care (WHO 2018). Due to the proven effect of EDCs on the development of endometriosis, special attention should be paid to these substances, and their use should be regulated and limited. Exposure to EDCs is widespread, but the knowledge among the population about the relationship between exposure to EDCs and disorders in reproductive health is limited (UNEP and WHO 2013). Emphasis on expanding to proper education of health care professionals, researchers, and the general population can help to reduce the exposure, the adverse health effects of these EDCs and incorporate safe management (Kahn et al. 2020, Kawa et al. 2021). Involvement of the health sector in chemical management would benefit the collection of information and the prevention of health effects. In particular, health risks from endocrine-disrupting chemicals could be reduced through more comprehensive assessments and testing methods that take health into account, with substantial socioeconomic savings for public health.

The studies were mostly well designed, using prospective cohorts, and the exposure assessment was based on the biomarker of exposure. Concerning the covariates and confounding affecting the endpoints, in most of the studies mentioned, confounders were included in data analysis. The manuscript was written based on PRISMA guidelines, but as only epidemiological studies were presented, there is no information about mechanism of the impact of selected chemicals on endometriosis. Nevertheless, this is the comprehensive review of the recently published epidemiologcal papers.

Future directions are needed to better understand the effects of EDCs, including the need to examine the effects of multiple and more consistent doses and to study different mechanisms of action. It is a challenge for future studies to overcome the experienced limitations. Such studies will be helpful in understanding how EDCs impact on endometriosis. By better understanding the impact of EDCs on endometriosis, we may be able to develop better policies for preventing EDC-induced toxicity. Understanding of the mechanisms may help us develop novel treatments for EDC-induced endometriosis and develop novel treatments for EDC-induced abnormalities.

## Conclusions

In conclusion, the results of the reviewed studies suggest that exposure to persistent endocrine disruptors may be associated with the occurrence of endometriosis (Table 1). The consistent results were found in the case of all described organochlorine pesticides (nine compounds), lead, PCB-28, PCB-138, PCB-153, PCB-180, PCB-201, 1,2,3,7,8 — PeCDD, and 2,3,4,7,8 — PeCDF. The results of the studies on mercury, cadmium, copper, chromium, zinc, PCB-52, PCB-101, PCB-118, PCB-138, PCB-156, PCB-180, 2,3,7,8 — TCDD, OCDF, PBB-77, PBB-101, PBB-153, PBB-180, PBDE-47, PBDE-183, PFOA, PFNA, PFBS, and PFOS exposure are inconclusive. Due to conflicting results further epidemiological studies are needed to confirm these findings, but reduction of exposure to minimize the effects induced by environmental pollutants is necessary.

## Supplementary Information

Below is the link to the electronic supplementary material.

Supplementary file1 (DOCX 107 KB)

Supplementary file2 (PDF 466 KB)

## Data Availability

Not applicable.

## References

[CR1] Alharbi OML, Basheer AA, Khattab RA, Ali I (2018). Health and environmental effects of persistent organic pollutants. J Mol Liq.

[CR2] Amir S, Shah STA, Mamoulakis C, Docea AO, Kalantzi OI, Zachariou A, Calina D, Carvalho F, Sofikitis N, Makrigiannakis A, Tsatsakis A (2021). Endocrine disruptors acting on estrogen and androgen pathways cause reproductive disorders through multiple mechanisms: a review. Int J Environ Res Public Health.

[CR3] Arnold SF, Vonier PM, Collins BM, Klotz DM, Guillette Jr LJ, McLachlan JA (1997). In vitro synergistic interaction of alligator and human estrogen receptors with combinations of environmental chemicals. Environ Health Perspect.

[CR4] Baltaci AK, Mogulkoc R, Baltaci SB (2019). Review: the role of zinc in the endocrine system. Pak J Pharm Sci.

[CR5] Bobb JF, Valeri L, Claus Henn B, Christiani DC, Wright RO, Mazumdar M, Godleski JJ, Coull BA (2015). Bayesian kernel machine regression for estimating the health effects of multi-pollutant mixtures. Biostatistics.

[CR6] Bordonné C, Puntonet J, Maitrot-Mantelet L, Bourdon M, Marcellin L, Dion E, Plu-Bureau G, Santulli P, Chapron C (2021). Imaging for evaluation of endometriosis and adenomyosis. Minerva Obstet Gynecol.

[CR7] Buck Louis et al., 2012 GM, Chen Z, Peterson CM, Hediger ML, Croughan MS, Sundaram R, Stanford JB, Varner MW, Fujimoto VY, Giudice LC, Trumble A, Parsons PJ, Kannan K (2012) Persistent lipophilic environmental chemicals and endometriosis: the ENDO Study. Environ Health Perspect 120(6): 811-816. 10.1289/ehp.1104432 10.1289/ehp.1104432 PMC3385438 22417635

[CR8] Cabrera-Rodríguez R, Luzardo OP, Almeida-González M, Boada LD, Zumbado M, Acosta-Dacal A, Rial-Berriel C, Henríquez-Hernández LA (2019). Association between prenatal exposure to multiple persistent organic pollutants (POPs) and growth indicators in newborns. Environ Res.

[CR9] Campbell S, Raza M, Pollack AZ (2016). Perfluoroalkyl substances and endometriosis in US women in NHANES 2003–2006. Reprod Toxicol.

[CR10] Chatman DL (1976). Endometriosis in the black woman. Am J Obstet Gynecol.

[CR11] Diamanti-Kandarakis E, Bourguignon JP, Giudice LC, Hauser R, Prins GS, Soto AM, Zoeller RT, Gore AC (2009). Endocrine-disrupting chemicals: an Endocrine Society scientific statement. Endocr Rev.

[CR12] Dutta S, Gorain B, Choudhury H, Roychoudhury S, Sengupta P (2022) Environmental and occupational exposure of metals and female reproductive health. Environ Sci Pollut Res Int 41:62067-62092. 10.1007/s11356-021-16581-934558053 10.1007/s11356-021-16581-9 34558053

[CR13] Egsmose EL, Bräuner EV, Frederiksen M, Mørck TA, Siersma VD, Hansen PW, Nielsen F, Grandjean P, Knudsen LE (2016). Associations between plasma concentrations of PCB 28 and possible indoor exposure sources in Danish school children and mothers. Environ Int.

[CR14] Endocrine Society (2022) 24 January 2022 https://www.endocrine.org/patient-engagement/endocrine-library/edcs

[CR15] Evich MG, Davis MJB, McCord JP, Acrey B, Awkerman JA, Knappe DRU, Lindstrom AB, Speth TF, Tebes-Stevens C, Strynar MJ, Wang Z, Weber EJ, Henderson WM, Washington JW (2022). Per- and polyfluoroalkyl substances in the environment. Science.

[CR16] Fernandez-Salguero PM, Hilbert DM, Rudikoff S, Ward JM, Gonzalez FJ (1996). Aryl-hydrocarbon receptor-deficient mice are resistant to 2,3,7,8-tetrachlorodibenzo-p-dioxin-induced toxicity. Toxicol Appl Pharmacol.

[CR17] Ferronato G, Viera MS, Prestes OD, Adaime MB, Zanella R (2018) Determination of organochlorine pesticides (OCPs) in breast milk from Rio Grande do Sul, Brazil, using a modified QuEChERS method and gas chromatography-negative chemical ionisation-mass spektrometry. Int J Environ Anal Chem 8(11):1005-1016. 10.1080/03067319.2018.1518441

[CR18] Fu Z, Xi S (2020). The effects of heavy metals on human metabolism. Toxicol Mech Methods.

[CR19] Gerkowicz SA, Curtis SW, Knight AK, Cobb DO, Spencer JB, Conneely KN, Terrell ML, Marcus M, Smith AK (2020). Endometriosis, endocrine disrupters, and epigenetics: an investigation into the complex interplay in women with polybrominated biphenyl exposure and endometriosis. J Assist Reprod Genet.

[CR20] Guillette Jr LJ (2006). Endocrine disrupting contaminants–beyond the dogma. Environ Health Perspect.

[CR21] Guo W, Pan B, Sakkiah S, Yavas G, Ge W, Zou W, Tong W, Hong H (2019). Persistent organic pollutants in food: contamination sources, health effects and detection methods. Int J Environ Res Public Health.

[CR22] Hammarstrand S, Jakobsson K, Andersson E, Xu Y, Li Y, Olovsson M, Andersson EM (2021). Perfluoroalkyl substances (PFAS) in drinking water and risk for polycystic ovarian syndrome, uterine leiomyoma, and endometriosis: a Swedish cohort study. Environ Int.

[CR23] Huang XM, Ma ST, Cui JT, Li P, Zeng XY, Yu ZQ (2017). Simultaneous determination of multiple persistent halogenated compounds in human breast milk. Chin J Anal Chem.

[CR24] Jackson LW, Howards PP, Wactawski-Wende J, Schisterman EF (2011). The association between cadmium, lead and mercury blood levels and reproductive hormones among healthy, premenopausal women. Hum Reprod.

[CR25] Jagić K, Dvoršćak M, Klinčić D (2021). Analysis of brominated flame retardants in the aquatic environment: a review. Arh Hig Rada Toksikol.

[CR26] Jaishankar M, Tseten T, Anbalagan N, Mathew BB, Beeregowda KN (2014). Toxicity, mechanism and health effects of some heavy metals. Interdiscip Toxicol.

[CR27] Kabata-Pendias A (2000) Trace elements in soils and plants (3rd ed.). CRC Press. 432. 10.1201/9781420039900

[CR28] Kahn LG, Philippat C, Nakayama SF, Slama R, Trasande L (2020). Endocrine-disrupting chemicals: implications for human health. Lancet Diabetes Endocrinol..

[CR29] Kato H, Fujii S, Takahashi M, Matsumoto M, Koizumi HM, Ono A, Hirose A (2015). Repeated dose and reproductive/developmental toxicity of perfluorododecanoic acid in rats. Environ Toxicol.

[CR30] Kawa IA, Masood A, Fatima Q, Mir SA, Jeelani H, Manzoor S, Rashid F (2021). Endocrine disrupting chemical bisphenol A and its potential effects on female health. Diabetes Metab Syndr May-Jun.

[CR31] Kelce WR, Gray LE (1998). Wilson EM Antiandrogens as environmental endocrine disruptors. Reprod Fertil Dev.

[CR32] Kim M, Kim SH, Kim HJ, Whang DH, Yun SC, Lee SR, Chae HD, Kang BM (2020). Plasma levels of polychlorinated biphenyl, genetic polymorphisms, and the risk of advanced stage endometriosis. Gynecol Endocrinol.

[CR33] Kim MG, Min YS, Ahn YS (2021). Does exposure of lead and cadmium affect the endometriosis?. Int J Environ Res Public Health.

[CR34] Knutsen H, Alexander J, Barregård L, Bignami M, Brüschweiler B, Ceccatelli S (2018). Risk to human health related to the presence of perfluorooctane sulfonic acid and perfluorooctanoic acid in food. EFSA J.

[CR35] Kranthi Kumar K, Uma Devi B, Neeraja P (2017). Integration of in silico approaches to determination of endocrine-disruptingperfluorinated chemicals binding potency with steroidogenic acute regulatory protein. Biochem Biophys Res Commun.

[CR36] Kristensen DM, Skalkam ML, Audouze K, Lesné L, Desdoits-Lethimonier C, Frederiksen H, Brunak S, Skakkebæk NE, Jégou B, Hansen JB, Junker S, Leffers H (2011). Many putative endocrine disruptors inhibit prostaglandin synthesis. Environ Health Perspect.

[CR37] Lai G-L, Yeh C-C, Yeh C-Y, Chen R-Y, Fu C-L, Chen C-H, Tzeng C-R (2017). Decreased zinc and increased lead blood levels are associated with endometriosis in Asian women. Reprod Toxicol.

[CR38] Le Magueresse-Battistoni B (2020). Adipose tissue and endocrine-disrupting chemicals: does sex matter?. Int J Environ Res Public Health.

[CR39] Lind PM, Lind L (2020). Are persistent organic pollutants linked to lipid abnormalities, atherosclerosis and cardiovascular disease? A review. J Lipid Atheroscler..

[CR40] Louis GM, Peterson CM, Chen Z, Hediger ML, Croughan MS, Sundaram R, Stanford JB, Fujimoto VY, Varner MW, Giudice LC, Kennedy A, Sun L, Wu Q, Kannan K (2012). Perfluorochemicals and endometriosis: the ENDO study. Epidemiology.

[CR41] Luebker DJ, Case MT, York RG, Moore JA, Hansen KJ, Butenhoff JL (2015). Two-generation reproduction and cross foster studies of perfluorooctanesulfonate (PFOS) in rats. Toxicology.

[CR42] Martínez-Zamora MA, Mattioli L, Parera J, Abad E, Coloma JL, van Babel B, Galceran MT, Balasch J, Carmona F (2015). Increased levels of dioxin-like substances in adipose tissue in patients with deep infiltrating endometriosis. Hum Reprod.

[CR43] Martyniuk CJ, Mehinto AC, Denslow ND (2020). Organochlorine pesticides: Agrochemicals with potent endocrine-disrupting properties in fish. Mol Cell Endocrinol.

[CR44] Matta K, Lefebvre T, Vigneau E, Cariou V, Marchand P, Guitton Y, Royer AL, Ploteau S, Le Bizec B, Antignac JP, Cano-Sancho G (2022). Associations between persistent organic pollutants and endometriosis: a multiblock approach integrating metabolic and cytokine profiling. Environ Int.

[CR45] Mier-Cabrera M, Jimenez-Zamudio L, Garcia-Latorre E, Cruz-Orozco O, Hernandez-Guerrero C (2010). Quantitative and qualitative peritoneal immune profiles, T-cell apoptosis and oxidative stress-associated characteristics in women with minimal and mild endometriosis. BJOG.

[CR46] Mitra P, Sharma S, Purohit P, Sharma P (2017). Clinical and molecular aspects of lead toxicity: an update. Crit Rev Clin Lab Sc.

[CR47] Miyawaki T, Hirakawa S, Todaka T, Hirakawa H, Hori T, Kajiwara J, Hirata T, Uchi H, Furue M (2015). A study on polychlorinated biphenyls specifically–accumulated in blood of Yusho patients collected from medical check-ups in 2012. Fukuoka Igaku Zasshi.

[CR48] Montano L, Pironti C, Pinto G, Ricciardi M, Buono A, Brogna C, Venier M, Piscopo M, Amoresano A, Motta O (2022). Polychlorinated biphenyls (PCBs) in the environment: occupational and exposure events, effects on human health and fertility. Toxics.

[CR49] Neblett MF, Curtis SW, Gerkowicz SA, Spencer JB, Terrell ML, Jiang VS, Marder ME, Barr DB, Marcus M, Smith AK (2020). Examining reproductive health outcomes in females exposed to polychlorinated biphenyl and polybrominated biphenyl. Sci Rep.

[CR50] Nieder R, Benbi DK, Reichl FX (2018) Microelements and their role in human health, soil components and human health. Springer, Dordrecht 317–374. 10.1007/978-94-024-1222-2

[CR51] Nisha G, Karapatti N, PydiSetty Y (2018). Recent advances in the bio-remediation of persistent organic pollutants and its effect on environment. J Clean Prod.

[CR52] Obaid F (2000) Toxicological profile for polychlorinated biphenyls (PCBs). Agency for Toxic Substances and Disease Registry, November U.S. Department of Health and Human Services, Public Health Service, Atlanta, GA 122–126 36888731

[CR53] OECD (2018) Toward a new comprehensive global database of per and polyfuoroalkyl substances (PFASs): summary on updating the OECD 2007 list of per-and polyfuoroalkyl substances (PFASs). 4 May 2018 http://www.oecd.org/officialdocuments/

[CR54] Ohoro CR, Adeniji AO, Okoh AI, Okoh OO (2021). Polybrominated diphenyl ethers in the environmental systems: a review. J Environ Health Sci Eng.

[CR55] Ota Y, Andou M, Ota I (2018). Laparoscopic surgery with urinary tract reconstruction and bowel endometriosis resection for deep infiltrating endometriosis. Asian J Endosc Surg.

[CR56] Ozga-Stachurska AM, Wójcik-Grudzień J, Pawłowska P, Rozenbajgier M (2022) The influence of dioxines on endometriosis development – study review. Journal of Education, Health and Sport [online]. 5 September 2022, T. 12, nr 9, s. 491–497 10.12775/JEHS.2022.12.09.057

[CR57] Papadopoulou E, Vafeiadi M, Agramunt S, Mathianaki K, Karakosta P, Spanaki A, Besselink H, Kiviranta H, Rantakokko P, Koutis A (2013). Maternal diet, prenatal exposure to dioxins and other persistent organic pollutants and anogenital distance in children. Sci Total Environ.

[CR58] Pastor Belda M, González-Franco JA, Rubio R, Campillo N, Hernández-Córdoba M, Torres C, Pérez-Cárceles MD, Viñas P (2021). Occurrence of organochlorine pesticides in human tissues assessed using a microextraction procedure and gas chromatography-mass spectrometry. J Anal Toxicol.

[CR59] Pérez-Debén S, Gonzalez-Martin R, Palomar A, Quiñonero A, Salsano S, Dominguez F (2020). Copper and lead exposures disturb reproductive features of primary endometrial stromal and epithelial cells. Reprod Toxicol.

[CR60] Pietroń WJ, Małagocki P (2017). Quantification of polybrominated diphenyl ethers (PBDEs) in food. A Review Talanta.

[CR61] Ploteau S, Antignac JP, Volteau C, Marchand P, Vénisseau A, Vacher V, Le Bizec B (2016). Distribution of persistent organic pollutants in serum, omental, and parietal adipose tissue of French women with deep infiltrating endometriosis and circulating versus stored ratio as new marker of exposure. Environ Int.

[CR62] Ploteau S, Cano-Sancho G, Volteau C, Legrand A, Vénisseau A, Vacher V, Marchand P, Le Bizec B, Antignac JP (2017). Associations between internal exposure levels of persistent organic pollutants in adipose tissue and deep infiltrating endometriosis with or without concurrent ovarian endometrioma. Environ Int.

[CR63] Pollack AZ, Buck Louis et al., 2012 GM, Chen Z, Petterson CM, Sundaram R, Croughan MS, Sum L, Hediger ML, Stanford JB, Varner MW, Palmer CD, Steuerwald AJ, Parsons PJ (2013) Trace elements and endometriosis: the ENDO study. Reprod Toxicol 42:41–48.10.1016/j.reprotox.2013.05.009 10.1016/j.reprotox.2013.05.009 PMC3836840 23892002

[CR64] Pollack AZ, Krall JR, Kannan K, Buck Louis et al., 2012 GM (2021) Adipose to serum ratio and mixtures of persistent organic pollutants in relation to endometriosis: findings from the ENDO Study. Environ Res 195:110732. 10.1016/j.envres.2021.110732 10.1016/j.envres.2021.110732 PMC8432300 33484721

[CR65] Rehman K, Fatima F, Waheed I, Hamid Akash MS (2018). Prevalence of exposure of heavy metals and their impact on health consequences. J Cell Biochem.

[CR66] Ren X, Zeng G, Tang L, Wang J, Wan Liu Y, Yu J, Yi H, Ye S (2018). Deng R Sorption, transport and biodegradation - an insight into bioavailability of persistent organic pollutants in soil. Sci Total Environ.

[CR67] Richardson JR, Fitsanakis V, Westerink RHS, Kanthasamy AG (2019). Neurotoxicity of pesticides. Acta Neuropathol.

[CR68] Roy A, Perkins NJ, Buck Louis et al., 2012 GM (2012) Assessing chemical mixtures and human health: use of Bayesian belief net analysis. J Environ Prot (Irvine, Calif) 3(6): 462-468. 10.4236/jep.2012.36056 10.4236/jep.2012.36056 PMC3484983 23125944

[CR69] Rumph JT, Stephens VR, Archibong AE, Osteen KG, Bruner-Tran KL (2020). Environmental endocrine disruptors and endometriosis. Adv Anat Embryol Cell Biol.

[CR70] Rzymski P, Tomczyk K, Rzymski P, Poniedziałek B, Opala T, Wilczak M (2015). Impact of heavy metals on the female reproductive system. Ann Agric Environ Med.

[CR71] Safe S, Wang F, Porter W, Duan R, McDougal A (1998). Ah receptor agonists as endocrine disruptors: antiestrogenic activity and mechanisms. Toxicol Lett.

[CR72] Sandra O (2016). Hormonal control of implantation. Ann Endocrinol (Paris).

[CR73] Schechter T, Finkelstein Y, Koren G (2005). Pregnant “DES daughters” and their offspring. Can Fam Physician.

[CR74] Silva N, Senanayake H, Waduge V (2013). Elevated levels of whole blood nickel in a group of Sri Lankan women with endometriosis: a case control study. BMC Res Notes.

[CR75] Smarr MM, Kannan K, Buck Louis et al., 2012 GM (2016) Endocrine disrupting chemicals and endometriosis. Fertil Steril 106(4): 959-966. 10.1016/j.fertnstert.2016.06.034 10.1016/j.fertnstert.2016.06.034 27424048

[CR76] Soave I, Caserta D, Wenger JM, Dessole S, Perino A, Marci R (2015). Environment and endometriosis: a toxic relationship. Eur Rev Med Pharmacol Sci.

[CR77] Sofo V, Götte M, Laganà AS, Salmeri FM, Triolo O, Sturlese E, Retto G, Alfa M, Granese R, Abrão MS (2015). Correlation between dioxin and endometriosis: an epigenetic route to unravel the pathogenesis of the disease. Arch Gynecol Obstet.

[CR78] Stephens VR, Rumph JT, Ameli S, Bruner-Tran KL, Osteen KG (2022) The potential relationship between environmental endocrine disruptor exposure and the development of endometriosis and adenomyosis. Front Physiol https://www.frontiersin.org/articles/10.3389/fphys.2021.807685 10.3389/fphys.2021.807685 PMC8832054 35153815

[CR79] Stillman RJ, Miller LC (1984). Diethylstilbestrol exposure in utero and endometriosis in infertile females. Fertil Steril.

[CR80] Tanrıkut E, Karaer A, Celik O, Celik E, Otlu B, Yilmaz E, Ozgul O (2014). Role of endometrial concentrations of heavy metals (cadmium, lead, mercury and arsenic) in the etiology of unexplained infertility. Eur J Obstet Gynecol Reprod Biol.

[CR81] Tricco AC, Lillie E, Zarin W, O'Brien KK (2018). PRISMA extension for scoping reviews (PRISMA-ScR): checklist and explanation. Ann Intern Med.

[CR82] UNECE (1998) Protocol to the 1979 convention on long-range transboundary air pollution on persistent organic pollutants; UNECE: Aarhus, Denmark. 24 June 1998 http://www.unece.org/env/lrtap/pops_h1.html

[CR83] United Nations Environment Programme (UNEP) (2013) WHO. State of the science of endocrine disrupting chemicals – 2012. An assessment of the state of the science of endocrine disruptors prepared by a group of experts for the United Nations Environment Programme (UNEP) and WHO, Geneva, Switzerland https://apps.who.int/iris/handle/10665/78102

[CR84] Upson K, De Roos AJ, Thompson ML, Sathyanarayana S, Scholes D, Barr DB, Holt VL (2013). Organochlorine pesticides and risk of endometriosis: findings from a population-based case-control study. Environ Health Perspect.

[CR85] Vested A, Giwercman A, Bonde JP, Gunnar T (2014). Persistent organic pollutants and male reproductive health. Asian J Androl.

[CR86] Vichi S, Medda E, Ingelido AM, Ferro A, Resta S, Porpora MG, Abballe A, Nisticò L, De Felip E, Gemma S, Testai E (2012). Glutathione transferase polymorphisms and risk of endometriosis associated with polychlorinated biphenyls exposure in Italian women: a gene-environment interaction. Fertil Steril.

[CR87] Vierke L, Berger U, Cousins IT (2013). Estimation of the acid dissociation constant of perfluoroalkyl carboxylic acids through an experimental investigation of their water-to-air transport. Environ Sci Technol.

[CR88] Wang B, Zhang R, Jin F, Lou H, Mao Y, Zhu W, Zhou W, Zhang P, Zhang J (2017). Perfluoroalkyl substances and endometriosis-related infertility in Chinese women. Environ Int.

[CR89] World Health Organization (WHO) 2018 International classification of diseases, 11th Revision (ICD-11) Geneva: WHO. 18 June 2018

[CR90] Yao M, Hu T, Wang Y, Du Y, Hu C, Wu R (2017). Polychlorinated biphenyls and its potential role in endometriosis. Environ Pollut.

[CR91] Yılmaz BK, Evliyaoğlu Ö, Yorgancı A, Özyer Ş, Üstün YE (2020). Serum concentrations of heavy metals in women with endometrial polyps. J Obstet Gynaecol.

[CR92] Zhang Y, Lu Y, Ma H, Xu Q, Wu X (2021). Combined exposure to multiple endocrine disruptors and uterine leiomyomata and endometriosis in US women. Front Endocrinol.

[CR93] Zhao B, Chuc Y, Hardyb D, Li XK, Ge RR (2010). Inhibition of 3_ and 17_-hydroxysteroid dehydrogenase activities in rat Leydig cells by perfluorooctane acid. J Steroid Biochem Mol Biol.

[CR94] Zoeller RT (2021). Endocrine disrupting chemicals and thyroid hormone action. Adv Pharmacol.

[CR95] Zondervan KT, Becker CM, Koga K, Missmer SA, Taylor RN, Viganò P (2018). Endometriosis. Nat Rev Dis Primers.

[CR96] Zondervan KT, Becker CM, Missmer SA (2020). Endometriosis. N Engl J Med.

