# Supplementary material for: An exposure to endocrine active persistent pollutants and endometriosis — a review of current epidemiological studies
Source: Environ Sci Pollut Res Int. 2022 Dec 23;30(6):13974–93. doi: 10.1007/s11356-022-24785-w (PMC9908711; doi:10.1007/s11356-022-24785-w)
Supplement: Supplementary file 2 — Supplementary file2 (PDF 466 KB) [file 11356_2022_24785_MOESM2_ESM.pdf]

Appendix

Environmental Science and Pollution Research

Review title: An exposure to endocrine active persistent pollutants and endometriosis - A review of current epidemiological studies.

Dorota Szczęsna[1]*, Katarzyna Wieczorek[1], Joanna Jurewicz[2]

1 Department of Chemical Safety; Nofer Institute of Occupational Medicine, St. Teresa street 8, 91-348 Lodz, Poland,

2 Department of Toxicology, Medical University of Lodz, Muszyńskiego 1A street, 90-151 Lodz, Poland

* Correspondence: dorota.szczesna@imp.lodz.pl phone number: +48426314703

Table of contents

Table S1. The association between mercury (Hg) and occurrence of endometriosis.

Table S2. The association between cadmium (Cd) and occurrence of endometriosis.

Table S3. The association between lead (Pb) and occurrence of endometriosis.

Table S4. The association between copper (Cu) and occurrence of endometriosis.

Table S5. The association between chromium (Cr) and occurrence of endometriosis.

Table S6. The association between Zinc (Zn) and occurrence of endometriosis.

Table S7. The association between nickel (Ni) and occurrence of endometriosis.

Table S8. The association between other metals: aluminum (Al), manganese (Mn) and iron (Fe) and occurrence of endometriosis.

Table S9. The association between organochlorine pesticides (OCPs) and occurrence of endometriosis.

Table S10. The association between polychlorinated biphenyls (PCBs) and occurrence of endometriosis..

Table S11. The association between polychlorinated dibenzo-p-dioxins and polychlorinated dibenzofurans and occurrence of endometriosis.

Table S12. The association between polybrominated biphenyls PBBs and occurrence of endometriosis.

Table S13. The association between polybrominated diphenyl ether (PBDEs) and occurrence of endometriosis.

Table S14. The association between per- and polyfluoroalkyls (PFAAs) and occurrence of endometriosis.

Table 1. The association between mercury (Hg) and occurrence of endometriosis.

EDCs	Study	Study design	Kind of samples/ technique	Concentration of endocrine disrupting chemicals (EDC)/ Detection Frequency (DF)	Study population	Results
Mercury (Hg)	Zhang Y., China, 2021 US population	Cross-sectional	whole blood/ CVAAS - cold vapor atomic absorption spectrophotometry	GM (Geometric mean): 0.89 µg/L<br>AM (Arithmetic mean): 1.39 µg/L<br>DF: 96.3%	Women (20 - 54 years) recruited from NHANES study 2001-2006:<br>Case group: 77<br>Control group: 1127<br>Endometriosis confirmed by questionnaire.	The relationship between EM and exposure to Hg was found in second tertile: OR: 2.77, 95% CI: 1.47-5.47<br>In comparison with the first tertile.
	Lai G.-L. Taiwan, 2017	Cross-sectional	blood/<br>ICP-MS – Inductively Coupled Plasma Mass Spectrometry	GM (Geometric mean):<br>Cases: 0.15µg/L<br>Controls: 0.10µg/L<br>LOD: 3x(SD) of double-deionized water for a minimum of 6 independent analyses<br>DF: 66.2%	190 infertile women including:<br>Case group: 68 patients with endometriosis<br>Control group:122 without endometriosis<br>Endometriosis diagnosis confirmed by laparoscopic surgery and also proved by pathology.	No significant association between EM and exposure to Hg - in second tertile:<br>OR_{Crude}: 0.64, 95% CI: 0.29, 1.41<br>$OR_{Adjusted}$: 0.66, 95% CI: 0.29, 1.54<br>in third tertile:<br>OR_{Crude}: 1.42, 95% CI: 0.71, 2.83<br>$OR_{Adjusted}$: 1.54, 95% CI: 0.72, 3.29<br>in comparison to the first tertile.
	Tanrıkut E Turkey, 2014	Case-control	Tissue (endometrial)/<br>AAS - Atomic Absorption Spectrometry	Concentration range: 0.6-65 µg/L<br>Median concentration: 0µg/L<br>LOD: 0.6 µg/L<br>DF: 0%	Women (18-40 years):<br>Case group: 33 women with unexplained infertility<br>Control group: 32 fertile women<br>Surgically confirmed endometriosis (endometrial biopsies).	OR_{Crude}: 0<br>$OR_{Adjusted}$: 0<br>by age, body mass index and endometrial heavy metal exposure<br>Hg was not detected in any endometrial samples from infertility and fertile women.
	Pollack A. Z. 2013 USA	Case-control	blood/urine<br>ICP-MS – Inductively Coupled Plasma Mass Spectrometry equipped	M (Mean):<br>blood<br>operative:<br>EM(190): 0.65µg/L<br>none(283):0.59µg/L	Women (18-44 yeras):<br>Cases: 473 operative cohort (EM=190, none=283)<br>Endometriosis confirmed by surgical visualization in	Blood<br>operative :<br>$OR_{Adjusted}$ = 1.22, 95% CI: 0.76, 1.94<br>population:

			with nebulizer and spray - chamber	population:<br>EM(14): 0.37μg/L<br>none(113):0.71μg/L<br>LOD: μg/dL<br>Urine<br>operative:<br>EM(190): 0.31μg/L<br>none(283):0.36μg/L<br>population:<br>EM(14): 0.43μg/L<br>none(113):0.48μg/L<br>LOD: μg/dL	operative (scheduled for laparoscopy and laparotomy)<br>Controls: 131 population cohort (EM=14, none=113)<br>Endometriosis confirmed by pelvic magnetic resonance imaging (MRI) in a population cohort.	$OR_{Adjusted}$ = 0.49, 955 CI: 0.11, 2.07<br>Urine<br>operative:<br>$OR_{Adjusted}$ = 0.92, 95% CI: 0.54, 1.55<br>population:<br>0.40, 95% CI: 0.06, 2.85

Table 2. The association between cadmium (Cd) and occurrence of endometriosis.

EDCs	Study	Study design	Kind of samples/ technique	Concentration of endocrine disrupting chemicals (EDC)/ Detection Frequency (DF)	Study population	Results
Cadmium (Cd)	Zhang Y., China, 2021<br>US population	Cross-sectional	whole blood/<br>ICP-MS – Inductively Coupled Plasma Mass Spectrometry	GM (Geometric mean): 0.39 μg/L<br>AM (Arithmetic mean): 0.53 μg/L<br>DF: 63.3%	Women (20 - 54 years) recruited from NHANES 2001-2006:<br>Case group: 77<br>Control group: 1127<br>Endometriosis confirmed by questionnaire.	Non-significant association between EM and exposure to Cd in second tertile and third tertile in comparison with the first tertile:<br>OR:1.29 (95% CI: 0.70, 2.42) and OR:1.00 (95% CI: 0.53, 1.92 - respectively)<br>In comparison with the first tertile.

	Kim M.-G.<br>Korea, 2021<br>Korea population	Cohort	Blood /<br>No information about technique of analysis.	No information about concentration.<br>LOD of BLL: 0.85 g/dL	Women (20-70 years) recruited from KNHICD study 2000-2005:<br>Case group: 26 542 female workers group who underwent a lead-associated special medical examination (BLLs: <5µg/dL and ≥5µg/dL)<br>Control group: noise-exposed worker group underwent noise-associated special medical examination (no information about number of participants)<br>Endometriosis confirmed by the principal's diagnosis.	Non-significant association between EM and exposure to Cd:<br>$OR_{Adjusted}$ (EM by BLLs): 1.88, 95% CI: 0.57-6.21; by age (>30, 30–39, and ≥40), cadmium, silica, organic solvent, and lead (<5µg/dL, ≥5µg/dL)<br><br>$OR_{Adjusted}$ (EM by Cd and Pb exposure): 1.90, 95% CI: 0.36, 10.04; by age (>30, 30–39, and ≥40), silica, organic solvent, lead, cadmium and lead and cadmium
	Tanrıkut E<br>Turkey, 2014	Case-control	Tissue (endometrial)/<br>AAS - Atomic Absorption Spectrometry	Median concentration: 19.58 µg/L<br>LOD: 0.01 µg/L<br>DF: 91%	Women (18-40 years):<br>Case group: 33 women with unexplained infertility<br>Control group: 32 fertile women<br>Surgically confirmed endometriosis (endometrial biopsies).	OR_{Crude}: 19.1, 95% CI: 4.7, 76.9<br>$OR_{Adjusted}$: 18.9, 95% CI: 4.5, 79.9 by age, body mass index and endometrial heavy metal exposure

	Silva N.<br>Sri Lanka, 2013	Case-control	whole blood/<br>GFAAS - Graphite Furnace Atomic Absorption Spectroscopy	GM (Geometric mean):<br>Cases: 0.7µg/L<br>Controls: 0.8µg/L<br>LOD: 0.01µg/L<br>DF: %	Women:<br>Case group: 50 (mean: 33 years)<br>Control group: 50 (mean: 32.7 years)<br>Endometriosis diagnosed visually - subsequent to laparotomy or laparoscopy	Geometric mean of metallo estrogens levels (µg/L):<br>Cases: 0.7, 95% CI: 0.7, 0.9<br>Controls: 0.8, 95% CI: 0.6, 1.0<br>Cases had lower levels as compared to controls but P value not statistically significant: 0.423
	Lai G.-L.<br>Taiwan, 2017	Cross-sectional	blood/<br>ICP-MS – Inductively Coupled Plasma Mass Spectrometry	GM (Geometric mean):<br>Cases: 0.42µg/L<br>Controls: 0.21µg/L<br>LOD: 0.02µg/L<br>DF: 75%	190 infertile women including:<br>Case group: 68 patients with endometriosis<br>Control group:122 without endometriosis<br>Endometriosis diagnosis confirmed by laparoscopic surgery and also proved by pathology.	Non-significant association between EM and exposure to Cd - in second tertile:<br>OR_{Crude}: 1.36, 95% CI: 0.63, 2.95<br>$OR_{Adjusted}$: 1.03, 95% CI: 0.45, 2.37<br>in third tertile:<br>OR_{Crude}: 1.90, 95% CI: 0.91, 3.95<br>$OR_{Adjusted}$: 1.73, 95% CI: 0.78, 3.92<br>in comparison to the first tertile.
	Pollack A. Z.<br>US, 2013	Case-control	blood/<br>ICP-MS – Inductively Coupled Plasma Mass Spectrometry equipped with nebulizer and spray-chamber	M (Mean):<br>operative:<br>EM(190):0.28µg/L<br>none(283):0.34µg/L<br>population:<br>EM(14): 0.25µg/L<br>none(113):0.30µg/L<br>LOD: 0.14µg/L	Women (18-44 yeras):<br>Cases: 473 operative cohort (EM=190, none=283)<br>Endometriosis confirmed by surgical visualization in operative (scheduled for laparoscopy and laparotomy)<br>Controls: 131 population cohort (EM=14, none=113)<br>Endometriosis confirmed by pelvic magnetic resonance imaging (MRI) in a population cohort.	Operative cohort: women with endometriosis versus unaffected women (geometric mean distributions of trace elements by endometriosis status irrespective of cohort):<br>OR: 0.28, 95% CI: 0.25, 0.31<br>OR: 0.34, 95% CI: 0.31, 0.37, respectively<br>Lowest tertile versus women in the highest tertile (correlations between metals Cd, Cr and Cu) :<br>$OR_{Adjusted}$= 0.52, 95% CI: 0.29, 0.93<br>Third versus first tertile (categorized into tertiles):

						$OR_{Adjusted}$=0.55, 95% CI: 0.31, 0.98 - diagnosis irrespective of statistical model

Table 3. The association between lead (Pb) and occurrence of endometriosis.

EDCs	Study	Study design	Kind of samples/ technique	Concentration of endocrine disrupting chemicals (EDC)/ Detection Frequency (DF)	Study population	Results
Lead (Pb)	Zhang Y., China, 2021 US population	Cross-sectional	whole blood/ ICP-MS – Inductively Coupled Plasma Mass Spectrometry	GM (Geometric mean): 1.00 µg/L AM (Arythmetic mean): 1.26 µg/L DF: 98 %	Women (20 - 54 years) recruited from NHANES study 2001-2006: Case group: 77 Control group: 1127 Endometriosis confirmed by questionnaire.	Non significant association between EM and exposure to Pb in second tertile and third tertile in comparison with the first tertile: $OR_{Adjusted}$: 0.65, 95% CI: 0.34, 1.22 and $OR_{Adjusted}$: 0.73, 95% CI: 0.37, 1.42; respectively)
	Kim M.-G. Korea, 2021 Korea population	Cohort	Blood / No information about technique of analysis.	No information about concentration. LOD of BLL: 0.85 g/dL	Women (20-70 years) recruited from KNHICD study 2000-2005: Case group: 26 542 female workers group who underwent a lead-associated special medical examination (BLLs: <5µg/dL and ≥5µg/dL)	Significant association between EM and exposure to Pb: $OR_{Adjusted}$ of EM by BLLs (<5µg/dL): 1.48, 95% CI: 1.11, 1.98*; *by age (>30, 30–39, and ≥40), cadmium, silica, organic solvent, and lead (<5µg/dL, ≥5µg/dL) and

					Control group: noise-exposed worker group underwent noise-associated special medical examination (no information about number of participants)<br>Endometriosis confirmed by the principal's diagnosis.	$OR_{Adjusted}$ (EM by Cd nad Pb exposure): 1.39, 95% CI: 1.14-1.67; by age (>30, 30–39, and ≥40), silica, organic solvent, lead, cadmium and lead and cadmium<br>Non significant association between EM and exposure to Pb:<br>$OR_{Adjusted}$ of EM by BLLs (≥5µg/dL): 1.31, 95% CI: 0.97, 1.76*
	Tanrıkut E<br>Turkey,<br>2014	Case-control	Tissue (endometrial)/<br>AAS - Atomic Absorption Spectrometry	Concentration range: 0.01-65 µg/L<br>Concentration - five cases of unexplained infertility: 0.12, 0.12, 0.24, 0.29 and 0.32µg/L<br>Concentration (average) - control group: 0.03µg/L<br>LOD: 0.01 µg/L<br>DF: 15.2%	Women (18-40 years):<br>Case group: 33 women with unexplained infertility<br>Control group: 32 fertile women<br>Surgically confirmed endometriosis (endometrial biopsies).	$OR_{Adjusted}$: 7.3, 95% CI: 0.8, 70.1 by age, body mass index and endometrial heavy metal exposure
	Silva N.<br>Sri Lanka,<br>2013	Case-control	whole blood/<br>TXRF - Total Reflection X-Ray Fluorescence	GM (Geometric mean):<br>Cases: 11.0µg/L<br>Controls: 6.9µg/L<br>LOD: 1.0µg/L<br>DF: %	Women:<br>Case group: 50 (mean: 33 years)<br>Control group: 50 (mean: 32.7 years)<br>Endometriosis diagnosed visually - subsequent to laparotomy or laparoscopy	Geometric mean of metallo estrogens levels (µg/L):<br>Cases: 11.0, 95% CI: 8.6, 13.3<br>Controls: 6.9, 95% CI: 5.7, 8.0<br>Cases had higher lead levels as compared to controls but P value not statistically significant: 0.389

	Lai G.-L.<br>Taiwan,<br>2017	Cross-sectional	blood/<br>ICP-MS – Inductively Coupled Plasma Mass Spectrometry	GM (Geometric mean):<br>Cases: 13.37µg/L<br>Controls: 8.53µg/L<br>LOD: 2µg/L<br>DF: 79.4%	190 infertile women including:<br>Case group: 68 patients with endometriosis<br>Control group:122 without endometriosis<br>Endometriosis diagnosis confirmed by laparoscopic surgery and also proved by pathology.	Non-significant association between EM and exposure to Pb in second tertile:<br>OR_{Crude}: 1.67, 95% CI: 0.78, 3.59<br>$OR_{Adjusted}$: 1.73, 95% CI: 0.77, 3.88<br>Significant association between EM and exposure to Pb in third tertile:<br>OR_{Crude}: 2.38, 95% CI: 1.07, 5.28<br>$OR_{Adjusted}$: 2.59, 95% CI: 1.11, 6.06<br>in comparison to the first tertile.<br>The lead level is associated with increasing odds.
	Pollack A. Z.<br>US, 2013	Case-control	blood/<br>ICP-MS – Inductively Coupled Plasma Mass Spectrometry equipped with nebulizer and spray - chamber	M (Mean):<br>operative:<br>EM(190): 0.61µg/L<br>none(283):0.67µg/L<br>population:<br>EM(14): 0.63µg/L<br>none(113):0.63µg/L<br>LOD: 0.05µg/dL	Women (18-44 years):<br>Cases: 473 operative cohort (EM=190, none=283)<br>Endometriosis confirmed by surgical visualization in operative (scheduled for laparoscopy and laparotomy)<br>Controls: 131 population cohort (EM=14, none=113)<br>Endometriosis confirmed by pelvic magnetic resonance imaging (MRI) in a population cohort.	Operative cohort: women with endometriosis versus unaffected women (geometric mean distributions of lead by endometriosis status irrespective of cohort): 0.61, 95% CI: 0.57, 0.66<br>0.67, 95% CI: 0.63, 0.71, respectively)<br>Third versus first tertile (categorized into tertiles):<br>$OR_{Adjusted}$= 0.84, 95% CI: 0.50, 1.41<br>- diagnosis irrespective of statistical model, not significant when adjusting for potential confounders

Table 4. The association between copper (Cu) and occurrence of endometriosis.

EDCs	Study	Study design	Kind of samples/ technique	Concentration of endocrine disrupting chemicals (EDC)/ Detection Frequency (DF)	Study population	Results

Copper (Cu)	Lai G.-L. Taiwan, 2017	Cross-sectional	blood/<br>ICP-MS – Inductively Coupled Plasma Mass Spectrometry	GM (Geometric mean):<br>Cases: 0.39mg/L<br>Controls: 0.48mg/L<br>LOD: 3x(SD) of double-deionized water for a minimum of 6 independent analyses<br>DF: 60.3%	190 infertile women including:<br>Case group: 68 patients with endometriosis<br>Control group:122 without endometriosis<br>Endometriosis diagnosis confirmed by laparoscopic surgery and also proved by pathology.	Non-significant association between EM and exposure to Cu -<br>in second tertile:<br>OR_{Crude}: 1.03, 95% CI: 0.51, 2.04<br>$OR_{Adjusted}$: 0.99, 95% CI: 0.47, 2.07<br>in third tertile:<br>OR_{Crude}: 0.52, 95% CI: 0.24, 1.13<br>$OR_{Adjusted}$: 0.45, 95% CI: 0.20, 1.02
	Pollack A. Z. US, 2013	Case-control	urine/<br>ICP-MS – Inductively Coupled Plasma Mass Spectrometry equipped with concentric quartz nebulizer and baffled quartz cyclonic spray chamber	M (Mean):<br>operative:<br>EM(190):10.64μg/L<br>none(283):10.33μg/L<br>population:<br>EM(14): 12.58μg/L<br>none(113):11.66μg/L<br>LOD: 3μg/L	Women (18-44 years):<br>Cases: 473 operative cohort (EM=190, none=283)<br>Endometriosis confirmed by surgical visualization in operative (scheduled for laparoscopy and laparotomy)<br>Controls: 131 population cohort (EM=14, none=113)<br>Endometriosis confirmed by pelvic magnetic resonance imaging (MRI) in a population cohort.	Third versus first tertile (categorized into tertiles):<br>$OR_{Adjusted}$=2.66, 95% CI: 1.26, 5.64
	Yılmaz B. K. Turkey, 2020	Case-control	Serum/<br>ICP-MS – Inductively Coupled Plasma Mass Spectrometry	Cu<br>Control:<br>M (Median): 119.00 μg/dL<br>Study:<br>M (Median): 114.00 μg/dL<br>Cu/Zn<br>Control:<br>M (Median): 1.295 μg/dL<br>Study:<br>M (Median): 114.00 μg/dL	Cases: 40 (44.9 ± 7.8 years)<br>Controls: 40 (45.4 ± 8.5 years)<br>Women with endometrial polyps as a single cause of abnormal bleeding according to the FIGO classification system was included in the study group.	No statistically significant differences were observed in terms of serum median levels of Cu:<br>Control: IQR: 38.75 Study:IQR: 29.00<br>The Cu/Zn ratio was significantly higher in the study group when compared with the control group:<br>Control: IQR: 0.547 Study:IQR: 0.442<br>There was no statistical

						difference in serum Cu levels with respect to the Cu IUD use history.

Table 5. The association between chromium (Cr) and occurrence of endometriosis.

EDCs	Study	Study design	Kind of samples/ technique	Concentration of endocrine disrupting chemicals (EDC)/ Detection Frequency (DF)	Study population	Results
Chromium (Cr)	Lai G.-L. Taiwan, 2017	Cross-sectional	blood/<br>ICP-MS – Inductively Coupled Plasma Mass Spectrometry	GM (Geometric mean):<br>Cases: 0.51µg/L<br>Controls: 0.56µg/L<br>LOD: 3x(SD) of double-deionized water for a minimum of 6 independent analyses<br>DF: 60.3%	190 infertile women including:<br>Case group: 68 patients with endometriosis<br>Control group:122 without endometriosis<br>Endometriosis diagnosis confirmed by laparoscopic surgery and also proved by pathology.	Non significant association between EM and exposure to Cr -<br>in second tertile:<br>OR_{Crude}: 0.74, 95% CI: 0.36, 1.53<br>$OR_{Adjusted}$: 0.78, 95% CI: 0.36, 1.68<br>in third tertile:<br>OR_{Crude}: 0.80, 95% CI: 0.39, 1.63<br>$OR_{Adjusted}$: 0.81, 95% CI: 0.38, 1.73
	Pollack A. Z. US, 2013	Case-control	urine/<br>ICP-MS – Inductively Coupled Plasma Mass Spectrometry equipped with concentric quartz nebulizer and baffled quartz cyclonic spray chamber	M (Mean):<br>operative:<br>EM(190):1.04µg/L<br>none(283):0.99µg/L<br>population:<br>EM(14): 1.26µg/L<br>none(113):1.01µg/L<br>LOD: 3µg/L	Women (18-44 years):<br>Cases: 473 operative cohort (EM=190, none=283)<br>Endometriosis confirmed by surgical visualization in operative (scheduled for laparoscopy and laparotomy)<br>Controls: 131 population cohort (EM=14, none=113)<br>Endometriosis confirmed by pelvic magnetic resonance imaging (MRI) in a population cohort.	Second versus first tertile (categorized into tertiles):<br>$OR_{Adjusted}$=1.97, 95% CI: 1.21, 3.19<br>Second versus lowest tertile (correlations between metals Cd, Cr and Cu):<br>$OR_{Adjusted}$=2.32, 95% CI: 1.42, 3.79<br>Urinary chromium reflected an increased odds.

Table 6. The association between Zinc (Zn) and occurrence of endometriosis.

EDCs	Study	Study design	Kind of samples/ technique	Concentration of endocrine disrupting chemicals (EDC)/ Detection Frequency (DF)	Study population	Results
Zinc (Zn)	Lai G.-L. Taiwan, 2017	Cross-sectional	blood/<br>ICP-MS – Inductively Coupled Plasma Mass Spectrometry	GM (Geometric mean):<br>Cases: 6.72mg/L<br>Controls: 11.86mg/L<br>LOD: three times the standard deviation(SD) of double-deionized water for a minimum of six independent analyses<br>DF: 45.6%	190 infertile women including:<br>Case group: 68 patients with endometriosis<br>Control group:122 without endometriosis<br>Endometriosis diagnosis confirmed by laparoscopic surgery and also proved by pathology.	Significant association between EM and exposure to Zn -<br>in second tertile:<br>OR_{Crude}: 0.47, 95% CI 0.23, 0.97<br>$OR_{Adjusted}$: 0.42, 95% CI 0.20, 0.92<br>in third tertile:<br>OR_{Crude}: 0.38, 95% CI: 0.18, 0.80<br>$OR_{Adjusted}$: 0.39, 95% CI: 0.18, 0.88<br>Zinc levels are associated with declining odds.
	Yılmaz B. K. Turkey, 2020	Case-control	Serum/<br>ICP-MS – Inductively Coupled Plasma Mass Spectrometry	Zn<br>Control:<br>M (Median): 0.84 µg/dL<br>Study:<br>M (Median): 0.75 µg/dL<br>Cu/Zn<br>Control:<br>M (Median): 1.295 µg/dL<br>Study:<br>M (Median): 114.00 µg/dL	Cases: 40 (44.9 ± 7.8 years)<br>Controls: 40 (45.4 ± 8.5 years)<br>Women with endometrial polyps as a single cause of abnormal bleeding according to the FIGO classification system was included in the study group.	No statistically significant differences were observed in terms of serum median levels of Zn:<br>Control: IQR: 0.18 Study:IQR: 0.09<br>The Cu/Zn ratio was significantly higher in the study group when compared with the control group:<br>Control: IQR: 0.547 Study:IQR: 0.442

Table 7. The association between nickel (Ni) and occurrence of endometriosis.

EDCs	Study	Study design	Kind of samples/ technique	Concentration of endocrine disrupting chemicals (EDC)/ Detection Frequency (DF)	Study population	Results

Nickel (Ni)	Silva N.<br>Sri Lanka, 2013	Case-control	whole blood/<br>TXRF - Total Reflection X-Ray Fluorescence (Ni, Pb)<br>GFAAS - Graphite Furnace Atomic Absorption Spectroscopy	GM (Geometric mean):<br>Cases: 2.6μg/L<br>Controls: 0.8μg/L<br>LOD: 0.05μg/L<br>DF: %	Women:<br>Case group: 50 (mean: 33 years)<br>Control group: 50 (mean: 32.7 years)<br>Endometriosis diagnosed visually - subsequent to laparotomy or laparoscopy	Geometric mean of metallo estrogens levels (μg/L) higher at cases as compared to control<br>P value: 0.016
	Yılmaz B. K.<br>Turkey, 2020	Case-control	Serum/<br>ICP-MS – Inductively Coupled Plasma Mass Spectrometry	Control:<br>M (Median): 1.86 μg/mL<br>Study:<br>M (Median): 1.22 μg/mL	Cases: 40 (44.9 ± 7.8 years)<br>Controls: 40 (45.4 ± 8.5 years)<br>Women with endometrial polyps as a single cause of abnormal bleeding according to the FIGO classification system was included in the study group.	The serum median levels of Ni were statistically lower in the study group when compared to the controls<br>Control: IQR: 2.67<br>Study: IQR: 1.17

Table 8. The association between other metals: aluminum (Al), manganese (Mn) and iron (Fe) and occurrence of endometriosis.

EDCs	Study	Study design	Kind of samples/ technique	Concentration of endocrine disrupting chemicals (EDC)/ Detection Frequency (DF)	Study population	Results
Aluminium (Al)	Yılmaz B. K.<br>Turkey, 2020	Case-control	Serum/<br>ICP-MS – Inductively Coupled Plasma Mass Spectrometry	Control:<br>M (Median): 9.47 μg/L<br>Study:<br>M (Median): 8.30 μg/L	Cases: 40 (44.9 ± 7.8 years)<br>Controls: 40 (45.4 ± 8.5 years)<br>Women with endometrial polyps as a single cause of abnormal bleeding according to the FIGO classification system was included in the study group.	The serum median levels of Al was statistically lower in the study group when compared to the controls<br>Control: IQR: 2.91<br>Study: IQR: 5.15

Manganese (Mn)	Lai G.-L. Taiwan, 2017	Cross-sectional	blood/ ICP-MS – Inductively Coupled Plasma Mass Spectrometry	GM (Geometric mean): Cases: 0.72µg/L Controls: 0.65µg/L LOD: 3x(SD) of double-deionized water for a minimum of 6 independent analyses DF: 60.3%	190 infertile women including: Case group: 68 patients with endometriosis Control group:122 without endometriosis Endometriosis diagnosis confirmed by laparoscopic surgery and also proved by pathology.	Non significant association between EM and exposure to Mn - in second tertile: OR_{Crude}: 0.68, 95% CI: 0.31, 1.50 $OR_{Adjusted}$: 0.68, 95% CI: 0.30, 1.56 in third tertile: OR_{Crude}: 1.24, 95% CI: 0.63, 2.44 $OR_{Adjusted}$: 1.25, 95% CI: 0.61, 2.57
Iron (Fe)	Lai G.-L. Taiwan, 2017	Cross-sectional	blood/ ICP-MS – Inductively Coupled Plasma Mass Spectrometry	GM (Geometric mean): Cases: 1185.86mg/L Controls: 1103.34mg/L LOD: 3x(SD) of double-deionized water for a minimum of 6 independent analyses DF: 63.2%	190 infertile women including: Case group: 68 patients with endometriosis Control group:122 without endometriosis Endometriosis diagnosis confirmed by laparoscopic surgery and also proved by pathology.	Non significant association between EM and exposure to Mn - in second tertile: OR_{Crude}: 0.74, 95% CI: 0.35, 1.56 $OR_{Adjusted}$: 0.70, 95% CI: 0.32, 1.54 in third tertile: OR_{Crude}: 1.00, 95% CI: 0.49, 2.02 $OR_{Adjusted}$: 0.83, 95% CI: 0.39, 1.76

Table 9. The association between organochlorine pesticides (OCPs) and occurrence of endometriosis.

EDCs	Study	Study design	Kind of samples/ Measurement technique of EDCs levels	Concentration of endocrine disrupting chemicals (EDC)/ Detection Frequency (DF)	Study population / Diagnostic	Results

B-hexachlor-cyklohexane (HCH)	Upson K. 2013<br>USA, western Washington State	Case-control (population-based)	serum/<br>GC/HRMS – Isotope dilution Gas Chromatography - High Resolution Mass Spectrometry	Median:<br>Cases: 51.91pg/g serum<br>Controls: 43.06pg/g serum<br>DF: LOD:10.0pg/g serum	Women (18 - 49 years) recruited from WREN study 1996-2001:<br>Case group: 248<br>Control group: 538 (past laparoscopy 47, no past laparoscopy 491 and infertility testing history 39, no infertility testing history 499).<br><br>Surgically confirmed endometriosis.	Comparison:<br>third vs. lowest quartile: OR = 1.7; 95% CI: 1.0, 2.8<br>highest vs. lowest quartile<br>$OR_{Adjusted}$ = 1.3, 95% CI: 0.8, 2.4<br>The association between serum β-HCH concentrations and endometriosis was stronger in analyses restricting cases to those with ovarian endometriosis:<br>third vs. lowest quartile:<br>$OR_{Adjusted}$ = 2.5; 95% CI: 1.5, 5.2<br>highest vs. lowest quartile:<br>$OR_{Adjusted}$ = 2.5; 95% CI: 1.1, 5.3
	Buck Louis G. M. 2012<br>USA, California, Utah, New York	Case-sectional matched cohort	serum/<br>GCHRMS - Electron capture detector Gas Chromatography – High Resolution Mass Spectrometry with	Median:<br>473 operative cohort EM: 0.0063ng/g serum<br>None: 0.0063ng/g serum<br>127 population EM: 0.066ng/g serum<br>None: 0.0063ng/g serum<br>LOD: 64%	Women (18–44 years) participating hospital surgical centers between 2007 and 2009:<br>Cases: 473 operative cohort (EM: 190, None: 283)Endometriosis confirmed using the gold standard of visualization and further qualified by histologic confirmation - scheduled for a laparoscopy or laparotomy.<br>22 women were excluded because their surgeries were canceled.<br>Controls: 127 population (EM:14, None:113)<br>Endometriosis confirmed by pelvic magnetic resonance imaging (MRI) - mainly ovarian endometriomas.<br>4 women were excluded because their MRIs were unreadable.	$OR_{Adjusted}$ = 1.72, 95% CI: 1.09, 2.72 – population cohort

	Ploteau S.<br>2017<br>France	Preliminary case-control	adipose tissue/<br>GC-HRMS - Gas Chromatography coupled to High-Resolution Mass Spectrometry on double sector instruments (JEOL MS) 700D and 800D) after electron impact ionization	Median:<br>Parietal adipose tissue: 15.585 pg/g lipid weight<br>Omental adipose tissue: 16.193 pg/g lipid weight<br>Detection rates: 100%. For lower detection rates, non-detected values were replaced<br>by limits of detection (LOD - upper bound approach).	Women (18 to 45 years)<br>Cases: 55<br>Controls: 44<br>enrolled during 2013 and 2015 in Pays de la Loire, France.<br>Surgical diagnosis of Deep Infiltrating Endometriosis (DIE) first based on clinical examination, of which 26 cases presented also OvE (Ovarian Endometriosis).	OR_{Crude} = 1.58, 95% CI: 1.03, 2.51<br>$OR_{Adjusted}$ = 1.58, 95% CI: 0.94, 2.80
	Pollack A. Z.<br>2021<br>USA	Case-control	Adipose-to-serum ratio (ASR)/<br>(GC/HRMS) - Gas Chromatography Mass Spectrometry with electron capture detector and high resolution MS	Median (interquartile range) concentrations for adipose/serum ratio (n=339):<br>Total Median (IQR): 10.7 (17.8)<br>EM (Median, IQR): 13.3, 26.9<br>None (MEdian, IQR): 8.5, 11.2<br>LOD:	Women (18-44 years) from the ENDO study (2007-2009):<br>Cases: 190<br>Controls: 283<br>Surgically-visualized incident endometriosis.	Single chemical regression models:<br>$OR_{Adjusted}$= 1.5, 95% CI: 1.2, 1.9<br>Chemical mixtures, compared quantiles of chemical class mixtures (comparing 75th percentile to 25th percentiles) for OCPs was<br>0.26, 95% PI: 0.05, 0.57<br>For the ASR, comparing 75th percentile to 25th percentiles for OCPs -<br>OR: 0.26, 95% PI: 0.05, 0.57<br>For adipose, comparing 75th percentile to<br>25th percentile for OCPs -<br>OR: 0.17, 95% PI: 0.21, 0.56.<br>For serum, comparing 75th percentile to 25th percentile for OCPs the change in estimate -<br>OR: 0.08, 95% PI: 0.36, 0.21

Mirex	Upson K. 2013<br>USA, western Washington State	Case-control (population-based)	serum/<br>GC/HRMS – Isotope dilution Gas Chromatography - High Resolution Mass Spectrometry	Median:<br>Cases: <10.00pg/g serum<br>Controls: <10.00pg/g serum<br>For categorizing exposures in the statistical analyses:<br>Controls: <15.47pg/g serum<br>DF:<br>LOD: 10.0pg/g serum – first quartile cutpoint	Women (18 - 49 years) recruited from WREN study 1996-2001:<br>Case group: 248<br>Control group: 538 (past laparoscopy 47, no past laparoscopy 491 and infertility testing history 39, no infertility testing history 499).<br><br>Surgically confirmed endometriosis.	Comparison:<br>highest vs. lowest category:<br>$OR_{Adjusted}$ = 1.5; 95% CI: 1.0, 2.2
γ-hexachloro-cyclohexane (γ-HCH)	Buck Louis G. M. 2012<br>USA, California, Utah, New York	Case-sectional m<br>atched cohort	Omental fat/<br>GCHRMS - Electron capture detector Gas Chromatography – High Resolution Mass Spectrometry with	Median:<br>339 operative cohort<br>EM: 0.1991ng/g fat<br>None: 0.1200ng/g fat<br>LOD: 0.060 (>12%)	Women (18–44 years) participating hospital surgical centers between 2007 and 2009:<br>Cases: 473 operative cohort (EM: 190, None: 283)Endometriosis confirmed using the gold standard of visualization and further qualified by histologic confirmation - scheduled for a laparoscopy or laparotomy.<br>22 women were excluded because their surgeries were canceled.<br><br>Controls: 127 population (EM:14, None:113)<br>Endometriosis confirmed by pelvic magnetic resonance imaging (MRI) - mainly ovarian endometriomas.<br>4 women were excluded because their MRIs were unreadable.	$OR_{Adjusted}$ = 1.27; 95% CI: 1.01, 1.59 – clinical, operative cohort

	Pollack A. Z.<br>2021<br>USA	Case-control	Adipose-to-serum ratio (ASR)/<br>(GC/HRMS) - Gas Chromatography Mass Spectrometry with electron capture detector and high resolution MS	Median (interquartile range) concentrations for adipose/serum ratio (n=339):<br>Total Median (IQR):6.9 (10.5)<br>EM (Median, IQR): 9.2, 17.5<br>None (MEdian, IQR): 6.0, 7.3<br>LOD:	Women (18-44 years) from the ENDO study (2007-2009):<br>Cases: 190<br>Controls: 283<br>Surgically-visualized incident endometriosis.	Single chemical regression models:<br>OR_{ASR}= 1.6, 95% CI:<br>1.2, 2.0<br>Chemical mixtures, compared quantiles of chemical class mixtures (comparing 75th percentile to 25th percentiles) for OCPs was<br>0.26, 95% PI: 0.05, 0.57<br>For the ASR, comparing 75th percentile to 25th percentiles for OCPs -<br>OR: 0.26, 95% PI: 0.05, 0.57.<br>For adipose, comparing 75th percentile to 25th percentile for OCPs -<br>OR: 0.17, 95% PI: 0.21, 0.56.<br>For serum, comparing<br>75th percentile to 25th percentile for OCPs -<br>OR: 0.08, 95% PI: 0.36, 0.21
Hexachlorocy klohexane (HCB)	Ploteau S.<br>2017<br>France	Preliminary case-control	adipose tissue/<br>GC-HRMS - Gas Chromatography coupled to High-Resolution Mass Spectrometry on double sector instruments (JEOL MS) 700D and 800D) after electron impact ionization	Median:<br>Parietal adipose tissue: 9.975 pg/g lipid weight<br>Omental adipose tissue: 11.046 pg/g lipid weight<br>Detection rates: 100%. For lower detection rates non-detected values were replaced	Women (18 to 45 years)<br>Cases: 55<br>Controls: 44<br>enrolled during 2013 and 2015 in Pays de la Loire, France.<br>Surgical diagnosis of Deep Infiltrating Endometriosis (DIE) first based on clinical examination, of which 26 cases	OR_{Crude} = 2.10, 95% CI: 1.31, 3.63<br>$OR_{Adjusted}$ = 2.06, 95% CI: 1.2, 3.91

				by limits of detection (LOD - upper bound approach).	presented also OvE (Ovarian Endometriosis).	
Trans-nonachlore	Ploteau S. 2017<br>France	Preliminary case-control	adipose tissue/<br>GC-HRMS - Gas Chromatography coupled to High-Resolution Mass Spectrometry on double sector instruments (JEOL MS) 700D and 800D) after electron impact ionization	Median:<br>Parietal adipose tissue: 4.266 pg/g lipid weight<br>Omental adipose tissue: 4.407 pg/g lipid weight<br>Detection rates: 100%. For lower detection rates non-detected values were replaced<br>by limits of detection (LOD - upper bound approach).	Women (18 to 45 years)<br>Cases: 55<br>Controls: 44<br>enrolled during 2013 and 2015 in Pays de la Loire, France.<br>Surgical diagnosis of Deep Infiltrating Endometriosis (DIE) first based on clinical examination, of which 26 cases presented also OvE (Ovarian Endometriosis).	$OR_{Adjusted}$ = 2.21, 95% CI: 1.24, 4.28
	Matta K. 2022<br>France	Pilot clinical-based	serum/<br>GC/HRMS – Gas Chromatography - High Resolution Mass Spectrometry	Medians and interquartile: ranges:<br>Control: (12) 1.53 (0.50 - 3.45)<br>noOMA (deep endometriosis without endometrioma): (26) 4.94 (3.86 - 6.84)<br>OMA (endometrioma): (49) 6.61 (5.25 - 9.59)	Case control study (EndoTox): 99 women (55 cases, 44 controls (2013–2015) and EndoxOmics (18-45 years) women n = 87 (2015-2018) resulting in a final pool of 186 women.<br>Case: 56<br>Controls: 130<br>Surgically confirmed endometriosis.	Pooled analysis:<br>$OR_{Adjusted}$: 3.38, 95% CI: 2.06, 5.98
Dieldrin	Ploteau S. 2017<br>France	Preliminary case-control	adipose tissue/<br>GC-HRMS - Gas Chromatography coupled to High-Resolution Mass Spectrometry on double sector instruments (JEOL MS) 700D and 800D) after electron impact ionization	Median:<br>Parietal adipose tissue: 5.521 pg/g lipid weight<br>Omental adipose tissue: 5.845 pg/g lipid weight<br>Detection rates: 100%. For lower detection rates non-detected values were replaced	Women (18 to 45 years)<br>Cases: 55<br>Controls: 44<br>enrolled during 2013 and 2015 in Pays de la Loire, France.<br>Surgical diagnosis of Deep Infiltrating Endometriosis (DIE) first based on clinical examination, of which 26 cases	$OR_{Adjusted}$ = 2.72, 95% CI: 1.57, 5.11

				by limits of detection (LOD - upper bound approach).	presented also OvE (Ovarian Endometriosis).	
Oxychlordane	Ploteau S. 2017 France	Preliminary case-control	adipose tissue/ GC-HRMS - Gas Chromatography coupled to High-Resolution Mass Spectrometry on double sector instruments (JEOL MS) 700D and 800D) after electron impact ionization	Median: Parietal adipose tissue: 5.096 pg/g lipid weight Omental adipose tissue: 6.219 pg/g lipid weight Detection rates: 100%. For lower detection rates non-detected values were replaced by limits of detection (LOD - upper bound approach).	Women (18 to 45 years) Cases: 55 Controls: 44 enrolled during 2013 and 2015 in Pays de la Loire, France. Surgical diagnosis of Deep Infiltrating Endometriosis (DIE) first based on clinical examination, of which 26 cases presented also OvE (Ovarian Endometriosis).	$OR_{Adjusted}$ = 3.22, 95% CI: 1.6, 7.7
Cis-heptachlore epoxyde	Ploteau S. 2017 France	Preliminary case-control	adipose tissue/ GC-HRMS - Gas Chromatography coupled to High-Resolution Mass Spectrometry on double sector instruments (JEOL MS) 700D and 800D) after electron impact ionization	Median: Parietal adipose tissue: 4.396 pg/g lipid weight Omental adipose tissue: 4.979 pg/g lipid weight Detection rates: 100%. For lower detection rates non-detected values were replaced by limits of detection (LOD - upper bound approach).	Women (18 to 45 years) Cases: 55 Controls: 44 enrolled during 2013 and 2015 in Pays de la Loire, France. Surgical diagnosis of Deep Infiltrating Endometriosis (DIE) first based on clinical examination, of which 26 cases presented also OvE (Ovarian Endometriosis).	$OR_{Adjusted}$ = 5.36, 95% CI: 2.44, 14.84
Total correlation: HCB, β-HCH, oxychlordane, cis-hept-epox, p,p′-DDE and dieldrin, trans-nonachlor, and p,p′-DDT	Ploteau S. 2016 France	Preliminary case-control	omental adipose tissue, parietal adipose tissue, and serum Gel permeation chromatography - (GPC)	Median: parietal and omental adipose tissues (ng/g l.w.): HCB 9.975; 11.046 b-HCH 15.585; 16.193 Trans-nonachlor 4.266; 4.407 Oxychlordane 5.096; 6.219 Cis-heptachlor epoxyde 4.396; 4.979	Women (18 to 45 years) Cases: 55 Controls: 45 enrolled during 2013 and 2015 in Pays de la Loire, France. Surgical diagnosis of Deep Infiltrating Endometriosis (DIE) first based on clinical examination, of which 26 cases	The correlation between the concentrations determined in omental and parietal adipose tissue appeared significant for OCPs

				Dieldrin 5.521; 5.845<br>p,p′-DDT 2.577; 100.0<br>p,p′-DDE 71.089; 80.797<br>The compounds weren't determined in serum.	presented also OvE (Ovarian Endometriosis). For all cases, magnetic resonance imaging (MRI) and surgery were performed. Control where consulting for other gynecological problem without endometriosis at the surgery and without any clinical symptoms of the disease.	

Table 10. The association between polychlorinated biphenyls (PCBs) and occurrence of endometriosis.

EDCs	Study	Study design	Kind of samples/ Measurement technique of EDCs levels	Concentration of endocrine disrupting chemicals (EDC)/ Detection Frequency (DF)	Study population / Diagnostic	Results
PCB-congeners 28<br>2,4,4'-Trichloro biphenyl	Buck Louis G. M. 2012<br>USA, California Utah, New York	Case-sectional matched cohort	omental fat/<br>GCHRMS - Electron capture detector Gas Chromatography – High Resolution Mass Spectrometry with	Median:<br>339 operative cohort EM: 0.1111ng/g<br>None: 0.1038ng/g<br>LOD: 0.065 (>37%)	Women (18–44 years) participating hospital surgical centers between 2007 and 2009:<br>Cases: 473 operative cohort (EM: 190, None: 283)Endometriosis confirmed using the gold standard of visualization and further qualified by histologic confirmation - scheduled for a laparoscopy or laparotomy.<br>Controls: 127 population (EM:14, None:113)<br>Endometriosis confirmed by pelvic magnetic resonance imaging (MRI) - mainly ovarian endometriomas.	OR_{Crude} = 1.30, 95 % CI: 1.04, 1.62 – operative cohort<br>$OR_{Adjusted}$ = 1.16, 95% CI: 0.92, 1.47 – operative cohort

PCB - congeners 74 2,4,4',5-Tetrachlorobiphenyl	Buck Louis G. M. 2012 USA, California Utah, New York	Case-sectional matched cohort	omental fat/ GCHRMS - Electron capture detector Gas Chromatography – High Resolution Mass Spectrometry with	Median: 339 operative cohort EM: 1.4303ng/g None: 1.8628ng/g LOD: 0.03 (>7%)	Women (18–44 years) participating hospital surgical centers between 2007 and 2009: Cases: 473 operative cohort (EM: 190, None: 283)Endometriosis confirmed using the gold standard of visualization and further qualified by histologic confirmation - scheduled for a laparoscopy or laparotomy. Controls: 127 population (EM:14, None:113) Endometriosis confirmed by pelvic magnetic resonance imaging (MRI) - mainly ovarian endometriomas.	OR_{Crude} = 0.77, 95 % 0.61, 0.96 – operative cohort $OR_{Adjusted}$ = 0.72, 95% CI: 0.55, 0.93 – operative cohort
PCB - congeners 151 2,2',3,5,5',6-Hexachlorobiphenyl	Buck Louis G. M. 2012 USA, California, Utah, New York	Case-sectional matched cohort	omental fat/ GCHRMS - Electron capture detector Gas Chromatography – High Resolution Mass Spectrometry with	Median: 339 operative cohort EM: 0.0500ng/g None: 0.0436ng/g LOD: 0.030 (>30%)	Women (18–44 years) participating hospital surgical centers between 2007 and 2009: Cases: 473 operative cohort (EM: 190, None: 283)Endometriosis confirmed using the gold standard of visualization and further qualified by histologic confirmation - scheduled for a laparoscopy or laparotomy. Controls: 127 population (EM:14, None:113) Endometriosis confirmed by pelvic magnetic resonance imaging (MRI) - mainly ovarian endometriomas.	OR_{Crude} = 1.31, 95% CI: 1.03, 1.67 – operative cohort $OR_{Adjusted}$ = 1.25, 95 % CI: 0.99, 1.56 – operative cohort

PCB - congeners 156 2,3,3',4,4',5-Hexachlorobiphenyl	Buck Louis G. M. 2012 USA, California, Utah, New York	Case-sectional matched cohort	omental fat/ GCHRMS - Electron capture detector Gas Chromatography – High Resolution Mass Spectrometry with	Median: 339 operative cohort EM: 0.3150ng/g None: 0.7038ng/g LOD: 0.030 (>20%)	Women (18–44 years) participating hospital surgical centers between 2007 and 2009: Cases: 473 operative cohort (EM: 190, None: 283)Endometriosis confirmed using the gold standard of visualization and further qualified by histologic confirmation - scheduled for a laparoscopy or laparotomy. Controls: 127 population (EM:14, None:113) Endometriosis confirmed by pelvic magnetic resonance imaging (MRI) - mainly ovarian endometriomas.	OR_{Crude} = 0.78, 95% CI: 0.62, 0.98 – operative cohort $OR_{Adjusted}$ = 0.74, 95 % CI: 0.57, 0.96 – operative cohort
	Martinez-Zamora M.A. 2015 Spain	Case-control	adipose tissues/ HRGC-HRMS - High-Resolution Gas Chromatography coupled with High-Resolution Mass Spectrometry	Mean: Cases: 3125.00 pg/g lipid Control: 2609.00 pg/g lipid	Women (18-40 years): Cases: 30 patients, Deep Infiltrating Endometriosis (DIE) group - after laparoscopic surgery, magnetic resonance imaging (MRI) and transvaginal sonography, confirmatory histological study for DIE after surgery Control group - 30 patients without any type of endometriosis	Toxic equivalence (TEQ) - PCB-156: DIE group: 0.10, 95% CI: 0.075, 0.20 Control group: 0.07, 95% CI: 0.03, 0.13 $OR_{Adjusted}$ = 3.26, 95% CI: 1.98, 6.15

PCB - congeners 201 2,2',3,3',4, 5',6,6'-Octachlorobiphenyl	Buck Louis G. M. 2012 USA, California, Utah, New York	Case-sectional matched cohort	omental fat/<br>GCHRMS - Electron capture detector Gas Chromatography – High Resolution Mass Spectrometry with	Median:<br>339 operative cohort EM: 0.1002ng/g<br>None: 0.0600ng/g<br>LOD: 0.030 (>16%)	Women (18–44 years) participating hospital surgical centers between 2007 and 2009:<br>Cases: 473 operative cohort (EM: 190, None: 283)Endometriosis confirmed using the gold standard of visualization and further qualified by histologic confirmation - scheduled for a laparoscopy or laparotomy.<br>Controls: 127 population (EM:14, None:113) Endometriosis confirmed by pelvic magnetic resonance imaging (MRI) - mainly ovarian endometriomas.	OR_{Crude} = 1.28, 95% CI: 1.03, 1.60 – operative cohort<br>$OR_{Adjusted}$ = 1.20, 95% CI: 0.94, 1.53 – operative cohort
PCB - congeners 201 2,2',3,3',4, 5',6,6'-Octachlorobiphenyl	Pollack A. Z. 2021 USA	Case-control	Adipose-to-serum ratio (ASR)/<br>(GC/HRMS) - Gas Chromatography Mass Spectrometry with electron capture detector and high resolution MS	Median (interquartile range) concentrations for adipose/serum ratio (n=339):<br>Total Median (IQR): 6.7 (15.9) (0.165)<br>EM (Median, IQR): 8.7 (22.6)<br>None (MEdian, IQR): 6.4 (13.2)<br>LOD:	Women (18-44 years) from the ENDO study (2007-2009):<br>Cases: 190<br>Controls: 283<br>Surgically-visualized incident endometriosis.	Single chemical regression models: associations for ASR of estrogenic PCBs with endometriosis were generally positive.<br>Comparison of single chemical odds ratios across serum, adipose and ASR: estrogenic PCBs, OR: 29%, 95% PI: 12%, 49%- adipose levels<br>OR: 36%, 95% PI: 18%, 56% - for ASR measures compared to serum levels.<br>Chemical mixtures:<br>For the ASR (comparing 75th percentile to 25th percentiles) for estrogenic PCBs:<br>OR: 0.37, 95% PI: 0.06, 0.80<br>For adipose, comparing 75th percentile to 25th percentile for estrogenic PCBs -<br>OR: 0.27, 95% PI: 0.18, 0.72<br>For serum, comparing<br>75th percentile to 25th percentile for estrogenic PCBs -

						OR: − 0.04, 95% PI: 0.2, 0.12
PCB - congeners 49 2,2',4,5'-Tetrachlorobiphenyl	Pollack A. Z. 2021 USA	Case-control	Adipose-to-serum ratio (ASR)/<br>(GC/HRMS) - Gas Chromatography Mass Spectrometry with electron capture detector and high resolution MS	Median (interquartile range) concentrations for adipose/serum ratio (n=339):<br>Total Median (IQR): 0.2 (0.4)<br>EM (Median, IQR): 0.3 (0.5)<br>None (MEdian, IQR): 0.2 (0.3)<br>LOD:	Women (18-44 years) from the ENDO study (2007-2009):<br>Cases: 190<br>Controls: 283<br>Surgically-visualized incident endometriosis.	Single chemical regression models: associations for ASR of estrogenic PCBs with endometriosis were generally positive.<br>Comparison of single chemical odds ratios across serum, adipose and ASR: estrogenic PCBs, OR: 29%, 95% PI: 12%, 49%- adipose levels<br>OR: 36%, 95% PI: 18%, 56% - for ASR measures compared to serum levels.<br>Chemical mixtures:<br>For the ASR (comparing 75th percentile to 25th percentiles) for estrogenic PCBs:<br>OR: 0.37, 95% PI: 0.06, 0.80<br>For adipose, comparing 75th percentile to 25th percentile for estrogenic PCBs -<br>OR: 0.27, 95% PI: 0.18, 0.72<br>For serum, comparing<br>75th percentile to 25th percentile for estrogenic PCBs -<br>OR: − 0.04, 95% PI: 0.2, 0.12

PCB - congeners 206 2,2',3,3',4,4',5,5',6-Nonachlorobiphenyl	Buck Louis G. M. 2012 USA, California, Utah, New York	Case-sectional matched cohort	serum/ GCHRMS - Electron capture detector Gas Chromatography – High Resolution Mass Spectrometry with	Median: 473 operative cohort EM: 0.0012ng/g serum None: 0.0016ng/g serum 127 population EM: 0.0027ng/g serum None: 0.0011ng/g serum LOD: 0.003 (>68%)	Women (18–44 years) participating hospital surgical centers between 2007 and 2009: Cases: 473 operative cohort (EM: 190, None: 283)Endometriosis confirmed using the gold standard of visualization and further qualified by histologic confirmation - scheduled for a laparoscopy or laparotomy. Controls: 127 population (EM:14, None:113) Endometriosis confirmed by pelvic magnetic resonance imaging (MRI) - mainly ovarian endometriomas.	OR_{Crude} = 0.79, 95% CI: 0.65, 0.95 – operative cohort OR_{Crude} = 1.10, 95% CI: 0.66, 1.83 – population cohort $OR_{Adjusted}$ = 0.79, 95% CI: 0.65, 0.97 – operative cohort $OR_{Adjusted}$ = 1.19, 95% CI: 0.72, 1.95 – population cohort
PCB - congeners 52 2,2',5,5'-Tetrachlorobiphenyl	Kim M. 2020 Korea	Case - control	plasma/ GC-MS - Gas Chromatography - Mass Spectrometry	Mean: Cases: 1.8 ng/g lipid Controls: 11.3 ng/g lipid LOD: 0.03-0.3 pg/µL	Women 160 (pelvic surgery) Cases: 61 women had surgical and histological evidence of advanced endometriosis Controls: 99 women	Comparison the highest tertile levels with the lowest tertile levels: OR_{Crude} = 0.328, 95% CI: 0.142, 0.759 $OR_{Adjusted}$ = 0.350, 95% CI: 0.138, 0.885
PCB - congeners 114 2,3,4,4',5-Pentachlorobiphenyl	Martinez-Zamora M.A. 2015 Spain	Case-control	adipose tissues/ HRGC-HRMS - High-Resolution Gas Chromatography coupled with High-Resolution Mass Spectrometry	Mean: Cases: 325 pg/g lipid Control: 202.50 pg/g lipid	Women (18-40 years): Cases: 30 patients, Deep Infiltrating Endometriosis (DIE) group - after laparoscopic surgery, magnetic resonance imaging (MRI) and transvaginal sonography, confirmatory histological study for DIE after surgery	Toxic equivalence (TEQ) - PCB-114: DIE group: 0.01 (0.008, 0.014) Control group: 0.006 (0.003, 0.013) $OR_{Adjusted}$ = 2.47, 95% CI: 1.24, 5.64 Total concentration - relationship between dioxin and deep infiltrating endometriosis (DIE): $OR_{Adjusted}$ = 2.24, 95% CI: 1.41, 7.31

					Control group - 30 patients without any type of endometriosis	
	Roy A., 2012 Baltimore, USA	Cohort	serum/<br>GC-EC - Gas Chromatography with Electron-Capture	Concentration range:<br>0.001 - 0.013 ng/g serum	Women (18–40 years) -<br>Cohort: 84<br>EM: 32<br>No EM: 52<br>Gold standard laparoscopic visualization (1999–2000).	$OR_{Adjusted}$ = 3.01, 95% CI: 2.25, 3.77*<br>comparisons are restricted to women in the 75th percentile for each congener relative to women in the 25th percentile
PCB - congeners 189 2,3,3',4,4',5,5'-Heptachlorobiphenyl	Martinez-Zamora M.A. 2015 Spain	Case-control	adipose tissues/<br>HRGC-HRMS - High-Resolution Gas Chromatography coupled with High-Resolution Mass Spectrometry	Mean:<br>Cases: 662.00 pg/g lipid<br>Control: 509.00 pg/g lipid	Women (18-40 years):<br>Cases: 30 patients, Deep Infiltrating Endometriosis (DIE) group - after laparoscopic surgery, magnetic resonance imaging (MRI) and transvaginal sonography, confirmatory histological study for DIE after surgery<br>Control group - 30 patients without any type of endometriosis	Toxic equivalence (TEQ) - PCB-189:<br>DIE group: 0.02 (0.014, 0.04)<br>Control group: 0.01 (0.003, 0.028)<br>$OR_{Adjusted}$ = 1.52. 95% CI: 1.09, 3.15<br>Total concentration - relationship between dioxin and deep infiltrating endometriosis (DIE)<br>$OR_{Adjusted}$ = 1.67, 95% CI: 1.16, 2.10

PCB - congeners 126 3,3',4,4',5-Pentachlorobiphenyl	Martinez-Zamora M.A. 2015 Spain	Case-control	adipose tissues/ HRGC-HRMS - High-Resolution Gas Chromatography coupled with High-Resolution Mass Spectrometry	Mean:<br>Cases: 29.30 pg/g lipid<br>Control: 24.50 pg/g lipid	Women (18-40 years):<br>Cases: 30 patients, Deep Infiltrating Endometriosis (DIE) group - after laparoscopic surgery, magnetic resonance imaging (MRI) and transvaginal sonography, confirmatory histological study for DIE after surgery<br>Control group - 30 patients without any type of endometriosis	Toxic equivalence (TEQ) - PCB-126:<br>DIE group: 3.10 (2.9, 4.7)<br>control group: 2.45 (1.41, 4.57)<br>$OR_{Adjusted}$ = 1.62, 95% CI: 1.21, 2.17<br>Total concentration - relationship between dioxin and deep infiltrating endometriosis (DIE):<br>$OR_{Adjusted}$ = 1.89, 95% CI: 1.21, 2.25
PCB - congeners 136 2,2',3,3',6,6'-Hexachlorobiphenyl	Roy A., 2012 Baltimore, USA	Cohort	serum/<br>GC-EC - Gas Chromatography with Electron-Capture	Concentration range:<br>0.001 - 0.013 ng/g serum	Women (18–40 years) -<br>Cohort: 84<br>EM: 32<br>No EM: 52<br>Gold standard laparoscopic visualization (1999–2000).	$OR_{Adjusted}$ = 1.79, 95% CI: 1.03, 2.55<br>comparisons are restricted to women in the 75th percentile for each congener relative to women in the 25th percentile
PCB - congeners 118, 138, 153, 180	Neblett II M. F., 2020 Georgia, USA	Cohort	serum/<br>GC-MS/MS - Gas Chromatography tandem Mass Spectrometry	Total PCB (ng/mL or ppb):<br>Geometric Mean: 0.43<br>Range: 0.03–2.60<br>Total PCB (ng/g lipids):<br>Geometric Mean: 61.44<br>Range: 4.46–517.48<br>LOD:<br>PCB-118: 1.4 pg/mL, PCB-138: 1.2 pg/mL, PCB-153: 1.6 pg/mL,<br>PCB-180: 0.7 pg/mL,	Women (18-59 years)<br>Cohort: 254 - serum PCB and PBB levels were measured<br>EM: 44 (17.3%)<br>Detailed questionnaire - endometriosis diagnosed by a doctor.	$OR_{Adjusted}$ = 1.02, 95% CI: 0.68, 1.53<br>no associations between serum PCB exposure and endometriosis

PCB - congeners 118, 153, 138, 170, 180	Vichi S.<br>2012<br>Italy	Case-control	serum/<br>HRGC-Ms/MS - High Resolution Gas Chromatography - Ion Trap tandem Mass Spectrometry	Geometric mean (ng/g fat)<br>Controls: 209.6<br>Cases: 301.3	Women (343),<br>Cases: 181<br>Controls: 162<br>Laparoscopic diagnosis and histologic confirmation of the presence of endometriosis. Determination<br>of serum concentrations of selected PCBs by ion-trap mass spectrometry (subgroup, 63 cases and 63 controls).	Total PCBs (Low ≤ 208 ng/g fat, medium 209–305 ng/g fat, high >305 ng/g fat):<br>medium: $OR_{Adjusted}$ = 4.22, 95% CI: 1.53, 11.6<br>high: $OR_{Adjusted}$ = 4.92, 95% CI: 1.78, 13.6<br>medium-high: 4.6, 95% CI: 1.9, 11.0 - versus low level (first tertile)
PCB congeners 118 2,3',4,4',5-Pentachlorobiphenyl				Geometric mean (ng/g fat):<br>Cases:<br>PCB 118: 24.1<br>Controls:<br>PCB 118: 15.8		PCB 118 (Low ≤ 13.2 ng/g fat, medium 13.3–24.2 ng/g fat, high >24.2 ng/g fat):<br>high: $OR_{Adjusted}$ = 3.18,95% CI: 1.26, 8.01<br>medium high: $OR_{Adjusted}$ = 2.62, 95% CI: 1.18, 5.83 - versus low level (first tertile)
PCB - congeners 118 2,3',4,4',5-Pentachlorobiphenyl	Kim M.<br>2020<br>Korea	Case - control	plasma/<br>GC-MS - Gas Chromatography - Mass Spectrometry	Mean:<br>Cases: 3.2 ng/g lipid<br>Controls: 5.9 ng/g lipid<br>LOD: 0.03-0.3 pg/µL	Women 160 (pelvic surgery)<br>Cases: 61 women had surgical and histological evidence of<br>advanced endometriosis<br>Controls: 99 women	Comparison the highest tertile levels with the<br>lowest tertile levels:<br>OR_{Crude} = 0.186, 95% CI: 0.079, 0.439<br>$OR_{Adjusted}$ = 0.320, 95% CI: 0.125, 0.817
PCB congeners 153 2,2',4,4',5,5'-Hexachlorobiphenyl	Vichi S.<br>2012<br>Italy	Case-control	serum/<br>HRGC-Ms/MS - High Resolution Gas Chromatography - Ion Trap tandem Mass Spectrometry	Geometric mean (ng/g fat)<br>Cases:<br>PCB 153: 96.3<br>Controls:<br>PCB 153: 61.9	Women (343),<br>Cases: 181<br>Controls: 162<br>Laparoscopic diagnosis and histologic confirmation of the presence of endometriosis. Determination	PCB 153 (Low ≤ 62 ng/g fat, medium 63–104 ng/g fat, high >104 ng/g fat):<br>medium: $OR_{Adjusted}$ = 3.97, 95% CI: 1.51, 10.5<br>high: $OR_{Adjusted}$ = 3.51, 95% CI: 1.37, 9.00<br>medium-high: $OR_{Adjusted}$ = 3.72, 95% CI: 1.63, 8.51 - versus low level (first tertile)

PCB congeners 138 2,2',3,4,4',5'-Hexachlorobiphenyl				Geometric mean (ng/g fat)<br>Cases<br>PCB 138: 48.8<br>Controls:<br>PCB 138: 32.8	of serum concentrations of selected PCBs by ion-trap mass spectrometry (subgroup, 63 cases and 63 controls).	PCB 138 (Low ≤ 33.6 ng/g fat, medium 33.7–56 ng/g fat, high >56 ng/g fat):<br>medium: $OR_{Adjusted}$ = 2.34, 95% CI: 1.00, 5.73<br>high: $OR_{Adjusted}$ = 3.22, 95% CI: 1.26,8.23<br>medium-high: $OR_{Adjusted}$ = 2.73, 95% CI: 1.24, 6.00- versus low level (first tertile)
PCB congeners 170 2,2',3,3',4,4',5-Heptachlorobiphenyl				Geometric mean (ng/g fat)<br>Cases<br>PCB 170: 9.3<br>Controls:<br>PCB 170: 6.7		PCB 170 (Low ≤ 5.37 ng/g fat, medium 5.38–12.4 ng/g fat, high >12.4 ng/g fat):<br>medium: $OR_{Adjusted}$ = 2.71, 95% CI: 1.05, 7.01<br>high: $OR_{Adjusted}$ = 2.94, 95% CI: 1.14, 7.57<br>medium-high: $OR_{Adjusted}$ = 2.83, 95% CI: 1.24, 6.48 - versus low level (first tertile)
PCB congeners 180 2,2',3,4,4',5,5'-Heptachlorobiphenyl				Geometric mean (ng/g fat)<br>Cases<br>PCB 180: 46.8<br>Controls:<br>PCB 180: 34.6		PCB 180 (Low ≤ 33.2 ng/g fat, medium 33.3–60.4 ng/g fat, high >60.4 ng/g fat):<br>high: $OR_{Adjusted}$ = 3.13, 95% CI: 1.28, 7.68<br>high: $OR_{Adjusted}$ = 2.41, 95% CI: 1.08, 5.37 - versus low/medium level (first/second tertile)
Total correlation: PCB 28;<br>52;<br>101 - (2,2',4,5,5'-Pentachlorobiphenyl(; 138;<br>153;<br>180;	Ploteau S.<br>2016<br>France	Preliminary case-control	omental adipose tissue, parietal adipose tissue, and serum<br>Gel permeation chromatography - (GPC)	Median: parietal, omental adipose and serum tissues (ng/g l.w.):<br>PCB 28: 1.01; 0.88; 0.92<br>PCB 52: 0.16; 0.19; 0.23<br>PCB 101: 0.37; 0.39; 0.32<br>PCB 138: 27.62; 30.48; 15.63<br>PCB 153: 58.90; 65.79; 35.50<br>PCB 180: 50.89; 54.93; 30.11	Women (18 to 45 years)<br>Cases: 55<br>Controls: 45<br>enrolled during 2013 and 2015 in Pays de la Loire, France.<br>Surgical diagnosis of Deep Infiltrating Endometriosis (DIE) first based on clinical examination, of which 26 cases presented also OvE (Ovarian Endometriosis). For all cases, magnetic resonance	The correlation between the concentrations determined in omental, parietal adipose tissue and serum appeared significant for PCBs.<br>Regarding non dioxin-like PCB:<br>WHO TEQ2005 dl-PCB value varied from 1.7 to 15.5 pg/g l.w. (median = 4.7 pg/g l.w.) in omental tissue, from 0.6 to 17.6 pg/g l.w. (median = 4.5 pg/g l.w.) in parietal adipose tissue, and from 1.2 to 10.1 pg/g l.w. (median = 3.5 pg/g l.w.) in serum.

				Sum 6 ndl-PCBs 137.06 ;147.88 ; 81.09	imaging (MRI) and surgery were performed. Control where consulting for other gynecological problem without endometriosis at the surgery and without any clinical symptoms of the disease.	Sum of 6 ndl-PCB (28, 52, 101, 138, 153, 180) varied from 35.0 to 632.6 ng/g l.w. (median = 147.9 ng/g l.w.) in omental tissue, from 18.3 to 625.9 ng/g l.w. (median = 137.1 ng/g l.w.) in parietal adipose tissue, and from 21.4 to 223.0 ng/g l.w. (median = 81.1 ng/g l.w.) in serum

Table 11. The association between polychlorinated dibenzo-p-dioxins and polychlorinated dibenzofurans and occurrence of endometriosis.

EDCs	Study	Study design	Kind of samples/ Measurement technique of EDCs levels	Concentration of endocrine disrupting chemicals (EDC)/ Detection Frequency (DF)	Study population / Diagnostic	Results
2,3,7,8 – TCDD 2,3,7,8-Tetrachlorodibenzodioxin	Martinez-Zamora M.A. 2015 Spain	Case-control	fasted adipose tissues from omentum/ HRGC-HRMS - High-Resolution Gas Chromatography coupled with High-Resolution Mass Spectrometry	Mean:<br>Cases: 0.70 pg/g lipid<br>Control: 0.40 pg/g lipid	Women (18-40 years):<br>Cases: 30 patients, Deep Infiltrating Endometriosis (DIE) group - after laparoscopic surgery, magnetic resonance imaging (MRI) and transvaginal sonography, confirmatory histological study for DIE after surgery<br>Control group - 30 patients without any type of endometriosis	Toxic equivalence (TEQ):<br>DIE group: 0.70 (0.53-0.76)<br>control group: 0.40 (0.32-0.64)<br>$OR_{Adjusted}$ = 1.41, 95% CI: 1.12, 2.10<br>Total concentration - relationship between dioxin and deep infiltrating endometriosis (DIE)<br>$OR_{Adjusted}$ = 1.41, 95% CI: 1.12, 2.10
	Ploteau S. 2017 France	Preliminary case-control	adipose tissue/ Chromatography coupled to High-Resolution Mass Spectrometry on double sector instruments (JEOL MS) 700D and 800D) after electron impact ionization	Median:<br>Parietal adipose tissue: 0.69 pg/g lipid weight<br>Omental adipose tissue: 0.75 pg/g lipid weight<br>Detection rates: 100%	Women (18 to 45 years)<br>Cases: 55<br>Controls: 44<br>enrolled during 2013 and 2015 in Pays de la Loire, France.<br>Surgical diagnosis of Deep Infiltrating Endometriosis (DIE) first based on clinical examination, of which 26	OR_{Crude} = 1.44, 95% CI: 0.96, 2.22<br>$OR_{Adjusted}$ = 1.65, 95% CI: 0.95, 3.02

					cases presented also OvE (Ovarian Endometriosis).	
1,2,3,7,8 – PeCDD 1,2,3,7,8-Pentachlorodibenzodioxin	Martinez-Zamora M.A. 2015 Spain	Case-control	fasted adipose tissues from omentum/ HRGC-HRMS - High-Resolution Gas Chromatography coupled with High-Resolution Mass Spectrometry	Mean:<br>Cases: 2.41 pg/g lipid<br>Control: 1.67 pg/g lipid	Women (18-40 years):<br>Cases: 30 patients, Deep Infiltrating Endometriosis (DIE) group - after laparoscopic surgery, magnetic resonance imaging (MRI) and transvaginal sonography, confirmatory histological study for DIE after surgery<br>Control group - 30 patients without any type of endometriosis	Toxic equivalence (TEQ):<br>DIE group: 2.41 (2.12, 2.89)<br>control group: 1.67 (1.11, 2.53)<br>$OR_{Adjusted}$ = 1.82, 95% CI: 1.36, 7.14<br>Total concentration - relationship between dioxin and deep infiltrating endometriosis (DIE)<br>$OR_{Adjusted}$ = 1.82, 95% CI: 1.36, 7.14
	Ploteau S. 2017 France	Preliminary case-control	adipose tissue/ Chromatography coupled to High-Resolution Mass Spectrometry on double sector instruments (JEOL MS) 700D and 800D) after electron impact ionization	Median:<br>Parietal adipose tissue: 2.47 pg/g lipid weight<br>Omental adipose tissue: 2.80 pg/g lipid weight<br>Detection rates: 100%	Women (18 to 45 years)<br>Cases: 55<br>Controls: 44<br>enrolled during 2013 and 2015 in Pays de la Loire, France.<br>Surgical diagnosis of Deep Infiltrating Endometriosis (DIE) first based on clinical examination, of which 26 cases presented also OvE (Ovarian Endometriosis).	OR_{Crude} = 1.76, 95% CI: 1.16, 2.78<br>$OR_{Adjusted}$ = 2.20, 95% CI: 1.23, 4.25

2,3,4,7,8 – PeCDF 2,3,4,7,8-Pentachlorodibenzofuran	Martinez-Zamora M.A. 2015 Spain	Case-control	fasted adipose tissues from omentum/ HRGC-HRMS - High-Resolution Gas Chromatography coupled with High-Resolution Mass Spectrometry	Mean:<br>Cases: 4.98 pg/g lipid<br>Control: 3.95 pg/g lipid	Women (18-40 years):<br>Cases: 30 patients, Deep Infiltrating Endometriosis (DIE) group - after laparoscopic surgery, magnetic resonance imaging (MRI) and transvaginal sonography, confirmatory histological study for DIE after surgery<br>Control group - 30 patients without any type of endometriosis	Toxic equivalence (TEQ):<br>DIE group: 1.55 (1.28, 1.85)<br>control group: 1.18 (0.84, 1.56)<br>$OR_{Adjusted}$ = 2.13, 05% CI: 1.97, 6.42)<br>Total concentration - relationship between dioxin and deep infiltrating endometriosis (DIE)<br>$OR_{Adjusted}$ = 1.94, 95% CI: 1.27, 5.16
	Ploteau S. 2017 France	Preliminary case-control	adipose tissue/ Chromatography coupled to High-Resolution Mass Spectrometry on double sector instruments (JEOL MS) 700D and 800D) after electron impact ionization		Women (18 to 45 years)<br>Cases: 55<br>Controls: 44<br>enrolled during 2013 and 2015 in Pays de la Loire, France.<br>Surgical diagnosis of Deep Infiltrating Endometriosis (DIE) first based on clinical examination, of which 26 cases presented also OvE (Ovarian Endometriosis).	Deep infiltrating endometriosis (DIE) vs Controls:<br>OR_{Crude} = 1.41, 95% CI: 0.94, 2.17<br>$OR_{Adjusted}$ = 1.41 95% CI: 0.82, 2.51<br>DIE with ovarian endometrioma (DIE +OvE) vs controls:<br>OR_{Crude} =2.16, 95% CI: 1.26, 4.03<br>$OR_{Adjusted}$ = 2.21, 95% CI: 1.06, 5.16
OCDF Octochlorodibenzofuran	Ploteau S. 2017 France	Preliminary case-control	adipose tissue/ Chromatography coupled to High-Resolution Mass Spectrometry on double sector instruments (JEOL MS) 700D and 800D) after electron impact ionization	Median:<br>Parietal adipose tissue: 0.55 pg/g lipid weight<br>Omental adipose tissue: 0.65 pg/g lipid weight<br>Detection rates: 100%. For lower detection rates non-detected values were replaced by limits of detection (LOD - upper bound approach).	Women (18 to 45 years)<br>Cases: 55<br>Controls: 44<br>enrolled during 2013 and 2015 in Pays de la Loire, France.<br>Surgical diagnosis of Deep Infiltrating Endometriosis (DIE) first based on clinical examination, of which 26 cases presented also OvE (Ovarian Endometriosis).	Deep infiltrating endometriosis (DIE) vs Controls:<br>OR_{Crude} = 4.72, 955 CI: 2.54, 10.06<br>$OR_{Adjusted}$ = 5.42, 95% CI: 2.73, 12.85<br>DIE with ovarian endometrioma (DIE +OvE) vs controls:<br>OR_{Crude} =5.40 (95% CI: 2.56, 14.16)<br>$OR_{Adjusted}$ =6.98 (95% CI: 2.94, 22.27)

	Martinez-Zamora M.A. 2015 Spain	Case-control	fasted adipose tissues from omentum/ HRGC-HRMS - High-Resolution Gas Chromatography coupled with High-Resolution Mass Spectrometry	Mean: Cases: 0.87 pg/g lipid Control: 0.65 pg/g lipid	Women (18-40 years): Cases: 30 patients, Deep Infiltrating Endometriosis (DIE) group - after laparoscopic surgery, magnetic resonance imaging (MRI) and transvaginal sonography, confirmatory histological study for DIE after surgery Control group - 30 patients without any type of endometriosis	No significant association with endometriosis risk. Toxic equivalence (TEQ): DIE group: 0.0003, 95% CI: 0.0002, 0.0005 control group: 0.0005, 95% CI: 0.0003, 0.0009 $OR_{Adjusted}$ = 0.84, 95% CI: 0.11, 1.43 Total concentration - relationship between dioxin and deep infiltrating endometriosis (DIE) $OR_{Adjusted}$ = 1.72, 95% CI: 1.16, 3.15

Table 12. The association between polybrominated biphenyls PBBs and occurrence of endometriosis.

EDCs	Study	Study design	Kind of samples/ Measurement technique of EDCs levels	Concentration of endocrine disrupting chemicals (EDC)/ Detection Frequency (DF)	Study population / Diagnostic	Results
PBB-congeners 77, 101, 153, 180 PBB-77: 3,3’,4,4’-tetrabromobiphenyl PBB-101: 2,2’,4,5,5’-pentabro	Neblett II M. F., 2020 Georgia, USA	Cross-sectional	serum/ GC-MS/MS - Gas Chromatography - tandem Mass Spectrometry	Total PBB (ng/mL or ppb): Geometric Mean: 0.10 Range: 0.01–4.96 Total PBB (ng/g lipids): Geometric Mean: 14.72 Range: 0.87–823.10 LOD: PBB-77: 4.5 pg/mL PBB-101: 3.9 pg/mL PBB-153: 2 pg/mL PBB-180: 5.6 pg/mL	Women (18-59 years) Cohort: 254 - serum PCB and PBB levels were measured EM: 44 (17.3%) Detailed questionnaire - endometriosis diagnosed by a doctor.	$OR_{Adjusted}$ = 1.04, 95% CI: 0.75, 1.43 – no associations between serum PBB exposure and endometriosis

mobiphenyl PBB-153: 2,2',4,4',5,5'-hexabromobiphenyl PBB-180: 2,2',3,3',4,4',5-heptabromobiphenyl	Gerkowicz S. A., 2020 Georgia, USA	Cross-sectional	serum/ GC-MS/MS - Gas Chromatography - tandem Mass Spectrometry	Total PBB (ng/mL): Geometric mean: EM: 0.43 No EM: 0.30 LOD: PBB-77: 4.5 pg/mL PBB-101: 3.9 pg/mL PBB-153: 2 pg/mL PBB-180: 5.6 pg/mL	Women (18-59 years) EM: 65 No EM: 240 Detailed questionnaire - endometriosis diagnosed by a doctor.	$OR_{Adjusted}$ = 0.97, 95% CI: 0.93, 0.99 39,877 CpG sites nominally are associated with endometriosis and PBB
PBB - congeners 153 2,2',4,4',5,5'-hexabromobiphenyl	Ploteau S. 2017 France	Preliminary case-control	adipose tissue/ GC-HRMS - Gas chromatography coupled to high-resolution mass spectrometry on double sector instruments (JEOL MS) 700D and 800D) after electron impact ionization	Median: Parietal adipose tissue: 0.21 pg/g lipid weight Omental adipose tissue: 0.24 pg/g lipid weight Detection rates: 100%. For lower detection rates non-detected values were replaced by limits of detection (LOD - upper bound approach).	Women (18 to 45 years) Cases: 55 Controls: 44 enrolled during 2013 and 2015 in Pays de la Loire, France. Surgical diagnosis of Deep Infiltrating Endometriosis (DIE) first based on clinical examination, of which 26 cases presented also OvE (Ovarian Endometriosis).	DIE vs controls (n=99) Deep infiltrating endometriosis (DIE) vs Controls: OR_{Crude} = 2.13, 95% CI: 1.31, 3.73 $OR_{Adjusted}$ = 3.91, 95% CI: 1.60-11.60 DIE with ovarian endometrioma (DIE +OvE) vs controls: OR_{Crude} =3.46 (95% CI: 1.74, 8.12) $OR_{Adjusted}$ =8.26 (95% CI: 2.27, 44.41)

Table 13. The association between polybrominated diphenyl ether (PBDEs) and occurrence of endometriosis.

EDCs	Study	Study design	Kind of samples/ Measurement technique of EDCs levels	Concentration of endocrine disrupting chemicals (EDC)/ Detection Frequency (DF)	Study population / Diagnostic	Results

PBDE-congeners 47 2,2’,4,4’-tetrabromodiphenyl ether	Buck Louis G. M. 2012 USA, California, Utah, New York	Case-sectional matched cohort	omental fat/ GCHRMS - Electron capture detector Gas Chromatography – High Resolution Mass Spectrometry with	Median:<br>339 operative cohort EM: 13.467ng/g<br>None: 21.589ng/g<br>LOD: 0.100 (>0%)	Women (18–44 years) participating hospital surgical centers between 2007 and 2009:<br>Cases: 473 operative cohort (EM: 190, None: 283)Endometriosis confirmed using the gold standard of visualization and further qualified by histologic confirmation - scheduled for a laparoscopy or laparotomy.<br>22 women were excluded because their surgeries were canceled.<br>Controls: 127 population (EM:14, None:113)<br>Endometriosis confirmed by pelvic magnetic resonance imaging (MRI) - mainly ovarian endometriomas.<br>4 women were excluded because their MRIs were unreadable.	$OR_{Adjusted}$ = 0.70, 95% CI: 0.55, 0.90 – operative cohort
PBDE-congeners 183 2,2’,3,4,4’,5’,6-heptabromodiphenyl ether	Buck Louis G. M. 2012 USA, California, Utah, New York	Case-sectional matched cohort	omental fat/ GCHRMS - Electron capture detector Gas Chromatography – High Resolution Mass Spectrometry with	Median:<br>339 operative cohort EM: 0.2317ng/g<br>None: 0.1118 ng/g<br>127 population<br>LOD: 1.200 (>68)%	Women (18–44 years) participating hospital surgical centers between 2007 and 2009:<br>Cases: 473 operative cohort (EM: 190, None: 283)Endometriosis confirmed using the gold standard of visualization and further qualified by histologic confirmation - scheduled for a laparoscopy or laparotomy.<br>22 women were excluded because their surgeries were canceled.<br>Controls: 127 population (EM:14, None:113)<br>Endometriosis confirmed by pelvic magnetic resonance imaging (MRI) - mainly ovarian endometriomas.	$OR_{Adjusted}$ = 1.21, 95% CI: 0.96, 1.52 – operative cohort

					4 women were excluded because their MRIs were unreadable.	
PBDE - congeners 183 2,2’,3,4,4’,5’,6-heptabromodiphenyl ether	Ploteau S. 2017 France	Preliminary case-control	adipose tissue/ Chromatography coupled to High-Resolution Mass Spectrometry on double sector instruments (JEOL MS) 700D and 800D) after electron impact ionization	Median:<br>Parietal adipose tissue: 0.18 pg/g lipid weight<br>Omental adipose tissue: 0.19 pg/g lipid weight<br>Detection rates: 100%. For lower detection rates non-detected values were replaced by limits of detection (LOD - upper bound approach).	Women (18 to 45 years)<br>Cases: 55<br>Controls: 44<br>enrolled during 2013 and 2015 in Pays de la Loire, France.<br>Surgical diagnosis of Deep Infiltrating Endometriosis (DIE) first based on clinical examination, of which 26 cases presented also OvE (Ovarian Endometriosis).	Deep infiltrating endometriosis (DIE) vs<br>Controls:<br>OR_{Crude} = 1.38, 95% CI: 0.92, 2.14<br>$OR_{Adjusted}$ = 1.64, 95% CI: 1.05, 2.71<br>DIE with ovarian endometrioma (DIE +OvE) vs controls:<br>OR_{Crude} =1.04 (95% CI: 0.63, 1.71)<br>$OR_{Adjusted}$ =1.29 (95% CI: 0.76, 2.30)
Total PBDEs - congeners 47; 99; 100; 153; 154; 209<br><br>PBDE-47: 2,2’,4,4’-tetrabromodiphenyl ether<br><br>PBDE-99: 2,2’,4,4’,5-pentabromodiphenyl ether<br><br>PBDE-100: 2,2’,4,4’,6-	Pollack A. Z. 2021 USA	Case-control	Adipose-to-serum ratio (ASR)/ (GC/HRMS) - Gas Chromatography Mass Spectrometry with electron capture detector and high resolution MS	Median (interquartile range) concentrations for adipose/serum ratio (n=339):<br>Congeners 47 (Median (IQR):<br>Total: 345.0 (493.1); EM: 314.6 (477.8); No EM: 363.6 (525.9) LOD:<br>Congeners 99 (Median (IQR):<br>Total: 363.2<br>(164.5); EM: 57.0 (105.6); No EM: 67.7 (223.5) LOD:<br>Congeners 100 (Median (IQR):<br>Total: 158.8 (335.9); EM: 124.6 (317.9); No EM: 190.6 (327.7)<br>LOD:	Women (18-44 years) from the ENDO study (2007-2009):<br>Cases: 190<br>Controls: 283<br>Surgically-visualized incident endometriosis.	Chemical mixtures:<br>for the ASR, comparing 75th percentile to 25th percentiles for PBDEs<br>OR: - 0.26, 95% PI: 0.05, 0.57<br>for adipose, comparing 75th percentile to<br>25th percentile for PBDEs<br>OR: 0.26, 95% PI: 0.58, 0.06<br>for serum, comparing 75th percentile to 25th percentile for PBDEs<br>OR: - 0.09, 95% PI: 0.38, 0.20<br>Comparison of single chemical odds ratios:<br>ASR compared to

pentabromodiphenyl ether<br><br>PBDE-153: 2,2',4,4',5,5'-hexabromodiphenyl ether<br><br>PBDE-154: 2,2',4,4',5,6'-hexabromodiphenyl ether<br><br>PBDE-209: decabromodiphenyl ether				Congeners 153 (Median (IQR):<br>Total: 332.6<br>(737.9); EM: 283.2 (714.9); No EM: 375.5 (757.8)<br>LOD:<br>Congeners 154 (Median (IQR):<br>Total: 14.5 (95.5); EM: 17.6 (112); No EM: 12.8 (87.7)<br>LOD:<br>Congeners 209 (Median (IQR):<br>Total: 38.0 (117.3); EM: 42.8 (101.3) ; No EM: 35.3 (147.1)<br>LOD:		serum levels - ORs for ASR of PBDEs were comparable regardless of biologic media.

Table 14. The association between per- and polyfluoroalkyls (PFAAs) and occurrence of endometriosis.

EDCs	Study	Study design	Kind of samples/ technique	Concentration of endocrine disrupting chemicals (EDC)/ Detection Frequency (DF)	Study population	Results

PFOA: Perfluoro-octanoic acid	Campbell S. 2016 USA	Case-control	serum/ LC/MS - Liquid Chromatography Mass Spectrometry	Geometric mean: EM (n=59): 3.48 (2.95, 4.11) ng/mL None (n=699): 2.84 (2.59, 3.13) LOD: 0.1 ng/mL LOD > 0.69%	Women n = 20 470 (20 and 50 years) Doctor-diagnosed endometriosis (n = 2493). Restricted to women with serum measurements of perfluo-roalkyl substances n = 753.	Q1(0.07-1.79): no data Q2 (1.80-2.69): $OR_{Adjusted}$: 1.07, 95% CI: 0.20, 5.78 Q3 (2.70-3.99): $OR_{Adjusted}$: 5.45, 95%1.19, 25.04 Q4 (4.00-20.60): $OR_{Adjusted}$: 2.86, 95% CI: 0.63, 12.91 ln-PFOA: $OR_{Adjusted}$: 1.33, 95%: 0.82, 2.17 n=650
PFOS: Perfluorooctane sulfonate		Case-control	serum/ LC/MS - Liquid Chromatography Mass Spectrometry	Geometric mean: EM (n=59): 16.28, 14.09, 18.81 ng/mL None (n=699): 13.36 (12,18. 14.66) LOD: 0.2-0.4 ng/mL LOD > 0.20%	Women n = 20 470 (20 and 50 years) Doctor-diagnosed endometriosis (n = 2493). Restricted to women with serum measurements of perfluo-roalkyl substances n = 753.	Q1(0.14-7.99): no data Q2 (8.00-12.29): $OR_{Adjusted}$: 1.89, 95% CI: 0.35, 10.17 Q3 (12.30-18.19): $OR_{Adjusted}$: 3.56, 95% 0.86, 14.74 Q4 (18.20-392.00): $OR_{Adjusted}$: 3.48, 95% CI: 1.00, 12.00 ln-PFOS: $OR_{Adjusted}$: 1.43, 95% CI: 0.88, 2.30 n=650
PFHxS: Perfluorohexane sulfonate		Case-control	serum/ LC/MS - Liquid Chromatography Mass Spectrometry	Geometric mean: EM (n=59): 1.31 (1.10, 1.58) ng/mL None (n=699): 1.23 (1.06, 1.41) LOD: 0.1-0.3 ng/mL LOD > 5.84%	Women n = 20 470 (20 and 50 years) Doctor-diagnosed endometriosis (n = 2493). Restricted to women with serum measurements of	Q1 (0.07-0.59): no data Q2 (0.60-1.19): $OR_{Adjusted}$: 1.74, 95% CI: 0.41, 7.35 Q3 (1.20-2.19): $OR_{Adjusted}$: 1.70, 95% CI: 0.57, 5.07 Q4 (2.20-19.40):

					perfluo-roalkyl substances n = 753.	$OR_{Adjusted}$: 1.47, 95% CI: 0.40, 5.41<br>ln-PFHxS: $OR_{Adjusted}$: 1.05, 95% CI: 0.79, 1.41<br>n=650
PFNA: Perfluorononanoic acid		Case-control	serum/ LC/MS - Liquid Chromatography Mass Spectrometry	Geometric mean:<br>EM (n=59): 1.00 (0.84, 1.19) ng/mL<br>None (n=699): 0.84 (0.74, 0.95)<br>LOD: 0.1 ng/mL<br>LOD > 1.98%	Women n = 20 470 (20 and 50 years) Doctor-diagnosed endometriosis (n = 2493).<br>Restricted to women with serum measurements of perfluo-roalkyl substances n = 753.	Q1 (0.07-0.59): no data<br>Q2 (0.60-0.79): $OR_{Adjusted}$: 3.76, 95% CI: 0.69, 20.66<br>Q3 (0.80-1.19): $OR_{Adjusted}$: 5.27, 95% CI: 1.20, 23.06<br>Q4 (1.20-15.40): $OR_{Adjusted}$: 3.24, 95% CI: 0.81, 12.91<br>ln-PFNA: $OR_{Adjusted}$: 1.22, 95% CI: 0.84, 1.78<br>n=650
PFASs:<br><br>PFHpS-perfluorohexane sulfonic acid, PFOS-perfluorooctane sulfonic acid, PFOA-perfluorooctanoic acid, PFNA, PFDA-perfluorodecanoic acid, PFUnA-Perfluoroundecanoic acid	Matta K. 2022 France	Pilot clinical-based	serum/ LC/HRMS – Liquid Chromatography - High Resolution Mass Spectrometry	Medians and interquartile ranges:<br>Control: (12);<br>No OMA (deep EM without endometrioma): (26);<br>OMA (endometrioma): (49)<br>PFHpS :<br>0.05 (0.05 - 0.07) ; 0.05 (0.05 - 0.13) ; 0.05 (0.05 - 0.12)<br>PFOS :	Case control study (EndoTox): 99 women (55 cases, 44 controls (2013–2015) and EndoxOmics (18-45 years) women n = 87 (2015-2018) resulting in a final pool of 186 women.<br>Case: 56<br>Controls: 130<br>Surgically confirmed endometriosis.	No relevant associations between PFAS and endometriosis, alone nor in mixtures.

				1.87 (1.24 - 2.32) ; 2.09 (1.56 - 3.38) ; 2.45 (1.65 - 3.44)<br>PFOA :<br>1.10 (0.77 - 1.69) ; 1.21 (0.81 - 1.58) ; 1.22 (0.81 - 1.58)<br>PFNA :<br>0.46 (0.31 - 0.52) ; 0.48 (0.37 - 0.67) ; 0.49 (0.41 - 0.65)<br>PFDA :<br>0.19 (0.17 - 0.25) ; 0.22 (0.18 - 0.33) ; 0.21 (0.18 - 0.32)<br>PFUnA :<br>0.13 (0.10 - 0.17) ; 0.15 (0.10 - 0.19) ; 0.13 (0.10 - 0.19)		
PFOA<br>Perfluoro-octanoic acid	Louis G.B.M<br>2012<br>US	Case-control	Serum/<br>LC/MSMS -Liquid Chromatography-Tandem Mass Spectrometry	Geometric mean [ng/mL]:<br>operative:<br>with EM (N=190): 2.65 none (N=283): 2.15<br>population<br>with EM (N=14): 2.49 none (N=113): 2.33	Women who were and were not seeking clinical care.<br>Operative: 495 women (18–44 years) scheduled for laparoscopy/laparotomy at one of 14 participating clinical sites in the Salt Lake City or San Francisco area, 2007–2009.<br>Population: 131 women age matched, residence within a 50-mile radius of participating clinics.<br>Endometriosis was defined based on	Adjusted for age (years) and body mass index (continuous) – operative samples:<br>PFOA: $OR_{Adjusted}$: 1.89, 95% CI: 1.17, 3.06<br>Adjusted for age (years), body mass index (continuous), and parity conditional on gravidity (never pregnant, pregnant without births, pregnant with births) – operative samples:<br>PFOA: $OR_{Adjusted}$: 1.62, 95% CI: 0.99, 2.66

					surgical visualization (in the operative sample) or magnetic resonance imaging (in the population sample).	
PFNA perfluorononanoic acid				Geometric mean [ng/mL]:<br>operative:<br>with EM (N=190): 0.69 none (N=283): 0.58<br>population<br>with EM (N=14): 0.71<br>none (N=113): 0.64		Adjusted for age (years) and body mass index (continuous) – operative samples:<br>PFNA: $OR_{Adjusted}$: 2.20, 95% CI: 1.02, 4.75<br>Adjusted for age (years), body mass index (continuous), and parity conditional on gravidity (never pregnant, pregnant without births, pregnant with births) – operative samples:<br>PFNA: $OR_{Adjusted}$: 1.99, 95% CI: 0.91, 4.33
PFBS perfluorobutane sulfo nic acid	Wang B. 2017 China	Case-control	Plasma/	Median:<br>1ng/mL	Cases: 157<br>Controls: 178 seeking infertility treatment because of male reproductive dysfunction in 2014 and 2015.<br>Endometriosis surgically confirmed	second vs. lowest tertile:<br>$OR_{Adjusted}$ = 3.74, 95% CI: 2.04, 6.84<br>highest vs. lowest tertile:<br>$OR_{Adjusted}$ = 3.04, 95% CI: 1.65, 5.57<br>The association restricted to subjects

						with no previous pregnancy:<br>second vs. lowest tertile:<br>$OR_{Adjusted}$ = 2.91, 95% CI: 1.28, 6.61<br>highest vs. lowest tertile:<br>$OR_{Adjusted}$ = 3.41, 95% CI: 1.52, 7.65)<br>The association restricted to subjects without other gynecologic pathology:<br>second vs. lowest tertile:<br>$OR_{Adjusted}$ = 4.65, 95% CI: 2.21, 9.82<br>highest vs. lowest tertile:<br>$OR_{Adjusted}$ = 3.36, 95% CI: 1.58, 7.15
PFAAs (total)<br>PFPeA<br>PFHxA<br>PFHpA<br>PFOA<br>PFNA<br>PFDA<br>PFUnDA<br>PFDoDA<br>PFBS<br>PFHxS<br>PFHpS<br>PFOS	Hammarstrand S., 2021<br>Swedish	Population - cohort	Serum/no information available	Median (range) ng/mL:<br>PFHxS<br>Never-high: 26.8 (0.1–974)<br>Ever-high: 194 (1.6–1,658)<br>Early-high: 37.9 (1.6–450)<br>Late-high: 243 (6.5–1,660)<br>Referencee: 0.9 (0.2–3.6)<br>PFOS<br>Never-high: 34.7 (0.6–838)	All females n =29,106 within the Ronneby Registry Cohort, having resided in Ronneby between 1985 and 2013 (area with water supply from the highly contaminated waterworks).<br>The whole cohort (29 106); ever high exposure at residency (7 823) and never high exposure at residency (21 283).	No increased HR for endometriosis was found<br>HR: 0.74, 95% CI 0.42, 1.29 - highest estimated PFAS

				Ever-high: 227 (4.5–1,555)<br>Early-high: 38.3 (4.5–467)<br>Late-highd: 279 (13.1–1,560)<br>Referencee: 4.3 (0.3–12.9)<br>PFOA<br>Never-high: 3.2 (0.04–48.8)<br>Ever-high: 12.7 (0.5–91.9)<br>Early-high: 2.9 (0.5–21.5)<br>Late-high: 15.2 (0.8–91.9)<br>Referencee: 1.6 (0.2–4.4)	Endometriosis according to the international classification ICD codes (20-50 years).	
